# Diversity of sugar-diphospholipid-utilizing glycosyltransferase families

**DOI:** 10.1038/s42003-024-05930-2

**Published:** 2024-03-07

**Authors:** Ida K. S. Meitil, Garry P. Gippert, Kristian Barrett, Cameron J. Hunt, Bernard Henrissat

**Affiliations:** 1 Department of Biotechnology and Biomedicine, Technical University of Denmark, Kgs. Lyngby, Denmark; 2 Architecture et Fonction des Macromolécules Biologiques, CNRS, Aix-Marseille Université, Marseille, France; 3 Department of Biological Sciences, King Abdulaziz University, Jeddah, Saudi Arabia

**Keywords:** Classification and taxonomy, Enzymes, Glycobiology, Carbohydrates

## Abstract

Peptidoglycan polymerases, enterobacterial common antigen polymerases, O-antigen ligases, and other bacterial polysaccharide polymerases (BP-Pols) are glycosyltransferases (GTs) that build bacterial surface polysaccharides. These integral membrane enzymes share the particularity of using diphospholipid-activated sugars and were previously missing in the carbohydrate-active enzymes database (CAZy; www.cazy.org). While the first three classes formed well-defined families of similar proteins, the sequences of BP-Pols were so diverse that a single family could not be built. To address this, we developed a new clustering method using a combination of a sequence similarity network and hidden Markov model comparisons. Overall, we have defined 17 new GT families including 14 of BP-Pols. We find that the reaction stereochemistry appears to be conserved in each of the defined BP-Pol families, and that the BP-Pols within the families transfer similar sugars even across Gram-negative and Gram-positive bacteria. Comparison of the new GT families reveals three clans of distantly related families, which also conserve the reaction stereochemistry.

## Abstract (web summary)

Glycosyltransferases that build bacterial surface polysaccharides were classified into 17 new CAZy families. A glycan analysis reveals that the enzymes within each family transfer similar sugars even across Gram-negative and Gram-positive bacteria.

## Introduction

Carbohydrate polymers (glycans) and glyco-conjugates are the most abundant biomolecules on Earth and adopt a wide range of functions including energy storage, structure, signaling, and mediators of host-pathogen interactions^1^. Due to the stereochemical diversity of monosaccharides and the many possible linkages they can engage into, glycans display an enormous structural diversity^2,3^. Yet, our knowledge on their assembly is far from complete, especially in comparison to the enzymes catalyzing their breakdown.

The transfer of sugar moieties to acceptor molecules such as proteins, lipids or other sugars, is catalyzed by enzymes called glycosyltransferases or GTs^4^. Campbell and colleagues proposed a sequence-based classification of GTs into 26 families^5^. The number of sequence-based families has since continued to grow based on the necessary presence of at least one experimentally characterized founding member to define a family, and is presented in the carbohydrate-active enzymes database (CAZy; www.cazy.org)^6^. An advantage of the sequence-based classification is that it readily enables genome mining for the presence of new family members. Today there are 118 GT families in the CAZy database and in contrast to the EC numbers^7^, the sequence-based classification implicitly incorporates the structural features of GTs including the conservation of the catalytic residues.

It was recognized very early that sequence-based GT families group together enzymes that can utilize different sugar donors and/or acceptors, illustrating how GTs can evolve to adopt novel substrates and form novel products^5,8^. Mechanistically, glycosyltransferases can be either retaining or inverting, based on the relative stereochemistry of the anomeric carbon of the sugar donor and of the formed glycosidic bond^4^. With almost no exceptions, this feature is conserved in previously defined sequence-based families, providing predictive power to this classification, as the orientation of the glycosidic bond can be predicted even if the precise transferred carbohydrate is not known.

The large majority of the 116 GT CAZy families use donors activated by nucleotide diphosphates. Eleven families utilize nucleotide monophospho-sugars (sialyl and KDO transferases), while 12 families utilize lipid monophospho-sugars. Until now, only one family in the CAZy database utilizes sugar-diphospholipid donors: the oligosaccharyltransferases of family GT66, which transfer a pre-assembled oligosaccharide to Asp residues for protein N-glycosylation^4,9^. Several sugar-diphospholipid-utilizing GTs are currently missing in the CAZy database, and here we classify new sugar-diphospholipid-utilizing GTs from four major functional classes that are all involved in the synthesis of bacterial cell wall polysaccharides.

The first of these four functional classes corresponds to the peptidoglycan polymerases, shape, elongation, division and sporulation (SEDS) proteins. These proteins polymerize peptidoglycan in complex with class B penicillin-binding proteins^10^. Several 3-D structures of SEDS proteins have been determined, and they harbor 10 transmembrane helices and one long extracellular loop^11–13^. This loop contains an Asp residue, which has been shown to be essential for SEDS function^11,14^.

The enzymes in the next two functional classes, bacterial polysaccharide polymerases (BP-Pol, also known as Wzy) and O-antigen ligase (O-Lig, also known as WaaL) are involved in the synthesis of lipopolysaccharides (LPS). LPS are polysaccharides on the membrane of Gram-negative bacteria, and consist of the highly diverse O-antigen attached to the Lipid A-core oligosaccharide located in the outer membrane^15^. The structure of the O-antigen determines the O-serotype of the bacteria. Most LPS structures are produced via the so-called Wzx/Wzy-dependent pathway^16,17^, for which the genes are located in a specific gene cluster^16^. In this pathway, BP-Pol catalyzes the polymerization of pre-assembled oligosaccharides attached to undecaprenyl pyrophosphate (Und-PP). Little is known about the activity of BP-Pols. Firstly, because they are difficult to express heterologously, and to date, only one study has demonstrated the activity of O-Pol in vitro^18^ and no experimentally determined 3-D structure is available. Secondly, because the sequences of BP-Pols are highly diverse with a sequence identity as low as 16% for different serotypes of the same species^16^, it is difficult to identify conserved residues. However, several studies have identified BP-Pols in the gene clusters of various species, paving the way for analyzing BP-Pol sequences across a large range of taxonomic origin (see below). These include some Gram-positive bacteria which also employ the Wzx/Wzy-dependent pathway to produce capsular polysaccharides, including *Streptococcus pneumoniae*^19^. The third functional class, O-Lig catalyzes the final step in the synthesis of LPS; the ligation of the newly synthesized polymer (O-antigen) onto Lipid A-core oligosaccharide^20^. A structure of O-Lig in complex with Und-PP has been reported, which showed a fold with 12 transmembrane helices and a long periplasmic loop containing several conserved residues; two Args which bind to the phosphates of Und-PP and a His which is proposed to activate the acceptor^21^.

The enzymes present in the fourth functional class, the enterobacterial common antigen polymerases (ECA-Pol, also known as WzyE) are involved in the synthesis of enterobacterial common antigen (ECA). In addition to the O-antigen, ECA is a specific polysaccharide that occurs on the cell surface in members of the Enterobacterales order. ECA consists of repeating units of N-acetylglucosamine, N-acetyl-D-mannosaminuronic acid and 4-acetamido-4,6-dideoxy-D-galactose^22^. ECA is also produced via the Wzx/Wzy-dependent pathway, where ECA-Pol performs the equivalent reaction to the BP-Pols^22^.

Structurally, the sugar-diphospholipid-utilizing GTs have an overall GT-C fold common to other integral membrane GTs, which is different from the globular nucleotide-sugar-utilizing GTs; GT-A and GT-B^4^. GT-C enzymes have a number of transmembrane helices that varies from 8 to 14^4,23^. Alexander and Locher recently suggested two subgroups of GT-C glycosyltransferases, GT-C_A_ and GT-C_B_, where O-Lig and SEDS make up GT-C_B_^23^. As no structures have been published of ECA-Pol and BP-Pols, these have not been assigned to a structural subgroup.

We have identified 17 new GT families covering a large number of the sugar-diphospholipid-utilizing GTs, by detailed analysis of the primary sequence of SEDS proteins, ECA-Pols, BP-Pols and O-Ligs. In addition, we examined how sequence diversity correlates with the diversity of the transferred oligosaccharides and with the stereochemical outcome of the glycosyl transfer reaction. The analysis also revealed that the new GT families organize in three clans across the functional classes suggestive of common ancestry. Despite of poor sequence alignments we manage to identify conserved potentially critical amino acids common within the clans.

## Results

### Peptidoglycan polymerases

For building the CAZy family of SEDS proteins, we used four characterized proteins as seed sequences: the proteins with PDB IDs 6BAR^11^, 8TJ3^13^ and 8BH1^12^, and the protein with GenBank accession CAB15838.1^24^. Family GT119 was created and initially populated by using BLAST against GenBank, and subsequently by searching against GenBank with a hidden Markov model (HMM) built from the retrieved sequences. GT119 is a very large family currently counting over 57,200 GenBank members in the CAZy database with a pairwise sequence identity of 19% over 221 residues for the most distant members. The taxonomic distribution of family GT119 follows what was reported in^14^, namely that this protein family is present in all bacteria except for Mycoplasma. It is present in most but not all planctomycetes.

For SEDS proteins, the glycosyl donor for the polymerization reaction is Lipid II (Und-PP-muropeptide, an activated disaccharide carrying a pentapeptide), where the Und-PP is α-linked. The carbohydrate repeat unit of peptidoglycan being β-linked, the glycosyl transfer reaction thus inverts the stereochemistry of the anomeric carbon involved in the newly formed glycosidic bond.

### Enterobacterial common antigen polymerases

The ECA-Pol which was studied by Maczuga et al.^25^ was used as seed sequence for building the ECA-Pol family. Although the CAZy database only lists GenBank entries^26^, we decided to build our multiple sequence alignments (MSAs) with sequences from the NCBI non-redundant database in order to capture more diversity. An ECA-Pol sequence library was thus constructed from the seed sequence using BLAST against the non-redundant database of the NCBI. The ECA-Pols were assigned to a single new CAZy family, GT120. To date this new family contains over 4800 GenBank members with high similarity (sequence identity greater than 38% over 414 residues), consistent with the conservation of acceptor, donor and product of the reaction.

As expected from their taxonomy-based designation, the ECA-Pol family (GT120) essentially contains sequences from the Enterobacterales order but also a few members of the Pasteurellales, suggesting that ECA-Pols of the latter were acquired by horizontal gene transfer. The ECA-Pol family uses a retaining mechanism, since the substrate repeat unit is axially linked to Und-PP and also axially linked in the final polymer.

### O-antigen ligases

With the aim of including the O-Ligs in the CAZy database, we collected 37 O-Lig sequences (Supplementary Data 1) and constructed a sequence library from these seed sequences using BLAST against the NCBI non-redundant database. A phylogenetic tree of the sequence library revealed four distantly related clades (Supplementary Fig. 1). The O-Ligs were included into one new CAZy family, GT121 with more than 16,700 members distributed in the four subfamilies.

The greater diversity of the GT121 O-Ligs compared to the GT119 peptidoglycan polymerases and GT120 ECA-Pol appears in the form of the four divergent clades in the O-Lig phylogenetic tree (Supplementary Fig. 1). We hypothesize that this increased diversity originates from the extensive donor and moderate acceptor variability of O-Ligs^15^. Taxonomically, the GT121 O-Lig family is present in most bacteria, including both Gram-negative and Gram-positive bacteria. The reaction performed by O-Ligs involves an inversion of the stereochemistry of the anomeric carbon since the sugar donor is axially bound to Und-PP and the reaction product is equatorially bound to Lipid A^20^.

A recently discovered O-Lig, WadA, is bimodular with a GT121 domain appended to a globular glycosyltransferase domain of family GT25, which adds the last sugar to the oligosaccharide core^27^. We have constructed a tree with representative WadA homologs from the GT121 family (Supplementary Fig. 2) and observe that most of the sequences appended to a GT25 domain form one clade in the tree, except for a few outliers. This suggests a coupled action of the GT25 and of the GT121 at least for the bimodular O-ligs and possibly for the entire family. The bimodular WadA O-Lig is observed in five genera including Mesorhizobium and Brucella.

### Other bacterial polysaccharide polymerases

The fourth functional class of Und-PP-sugar-utilizing GTs are the BP-Pols. As previously mentioned, there is only one experimentally characterized BP-Pol^18^, but several studies have identified BP-Pols from the polysaccharide gene clusters, and we decided to build our families based on these published reports. We thus collected 363 predicted BP-Pol sequences from seven studies for various species, both Gram-negative and Gram-positive bacteria: *Escherichia coli*^28^, *Shigella boydii*, *Shigella dysenteriae*, *Shigella flexneri*^29^, *Salmonella enterica*^30^, *Yersinia pseudotuberculosis*, *Yersinia similis*^31^, *Pseudomonas aeruginosa*^16^, *Acinetobacter baumanii*, *Acinetobacter nosocomialis*^32^ and *Streptococcus pneumoniae*^19^ (Supplementary Data 2).

In contrast to ECA-Pols, the donors as well as the acceptors of BP-Pols are highly variable. Others have reported an exceptional sequence diversity of BP-Pols even within the same species^16^. We also found that the sequences of BP-Pols are extremely diverse, and global alignments failed to reveal any conserved residue due to both sequence diversity and to the difficulty in aligning proteins with multiple and variable numbers of transmembrane helices. It was therefore not possible to build a single family that could capture the diversity of BP-Pols.

In order to group BP-Pols into similarity clusters that we could include as families in the CAZy database, we first built a sequence library by running BLAST against the NCBI non-redundant database for each of the 363 BP-Pol seeds. Clustering of the BP-Pols proved challenging. A phylogenetic analysis was not possible because of their great diversity, and a sequence similarity network (SSN) analysis alone would either result in very small clusters (using a strict threshold) or larger clusters that were linked because of insignificant relatedness (using a loose threshold).

**Fig. 1:**
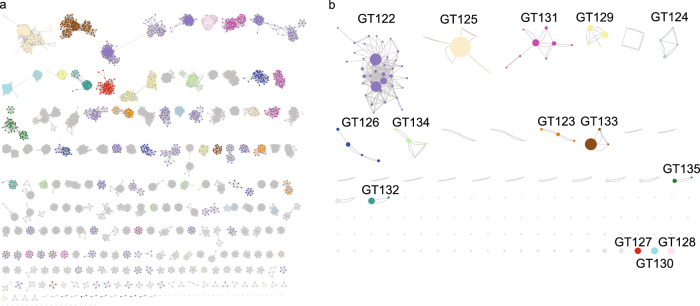
Clustering of BP-Pol sequences. **a** The first step of the clustering; SSN network with nodes representing individual proteins and edges representing pairwise alignment bit scores. Proteins are linked by edges if they have a pairwise score above 110. The resulting clusters are sorted according to number of protein members, with the largest cluster in the upper left corner. **b** The second step of the clustering: HMM models were built for each SSN cluster and the HMMs were compared using HHblits. A network was built with nodes representing SSN clusters and edges representing HHblits scores. SSN clusters are linked by edges if they have an HHblits score higher than 160. The resulting clusters are referred to as superclusters and are sorted according to number of SSN clusters. There are two edges between nodes, when the HHblits score is above 160 in both directions. The size of the nodes represents the number of members in the SSN cluster. The 14 largest superclusters (>150 GenBank members) define CAZy families GT122 - GT135. Nodes are colored consistently according to their respective CAZy family in both (**a**, **b**).

Instead, we used a combination of SSN and HMM comparisons: First, we used an SSN with a strict threshold which would allow us to build good MSAs for the resulting clusters. This resulted in 204 clusters (Fig. 1a). Next, we created an HMM profile of each SSN cluster and compared the HMMs by all-vs-all pairwise HHblits, a program that aligns two HMMs and calculates a similarity score^33^. We then combined the SSN clusters into superclusters in a network analysis based on the HHblits scores (Fig. 1b), resulting in 27 superclusters of varying sizes and 86 singleton clusters. Interestingly, the BP-Pols clustered across taxonomy, and even BP-Pols from Gram-positive and Gram-negative bacteria clustered together. The 14 largest superclusters define new GT families in the CAZy database (GT122-GT135) with a number of members ranging from 159 to 5979 at the time of submission. Only 150 of the 363 original seeds are included in the new families. We thus expect that many more BP-Pol families will be created in the future, as the amount and diversity of data increase.

All of the BP-Pol families are present in a wide taxonomic range, and outside of the taxonomic orders of the original seeds. Several of the families contain members from both Gram-positive and Gram-negative bacteria, for example GT122, GT130, and GT134.

As a way of evaluating our families, we performed structural superimpositions of AlphaFold models of distantly related members of each family. As an example, superimpositions of five distantly related members of GT122 are shown in Supplementary Fig. 3. The sequence identity between these members is relatively low (between 21.4 and 24.3%). Yet, they still produce a meaningful superimposition, and notably, the conserved residues are oriented very similarly.

### Analyzing the sugars transferred by bacterial polysaccharide polymerases

Next, we investigated how the BP-Pol families relate to the structures of the transferred oligosaccharide repeat units. We retrieved the serotype-specific sugar structures, which were reported in the review papers^16,19,29–32,34^. Additionally, nine sugar structures were included, which were published after the review papers^35–39^. Out of the 150 BP-Pol seed sequences that were included in the new CAZy families, we matched 131 with a sugar structure. The repeat units are oligosaccharides with 3–7 monomers within the backbone, often with branches. In most of the cases, the bond which is formed by the polymerase has been identified in the review papers based on the other GTs in the gene cluster which assemble the repeat units.

Having retrieved the sugar structures, we first analyzed the stereochemistry of the bond catalyzed by the polymerase. As mentioned above, the stereochemical mechanism (inverting or retaining) is usually well conserved in the CAZy GT families. The repeat unit structures are always axially linked (*α* for D-sugars and *β* for L-sugars) to the Und-PP moiety before polymerization. There are two possible mechanisms for the BP-Pol-catalyzed polymerization reaction, either retaining or inverting the axial configuration. Thus, if the bond formed by the polymerase is axial, the mechanism is retaining and if the bond formed by the polymerase is equatorial, the mechanism is inverting.

**Fig. 2:**
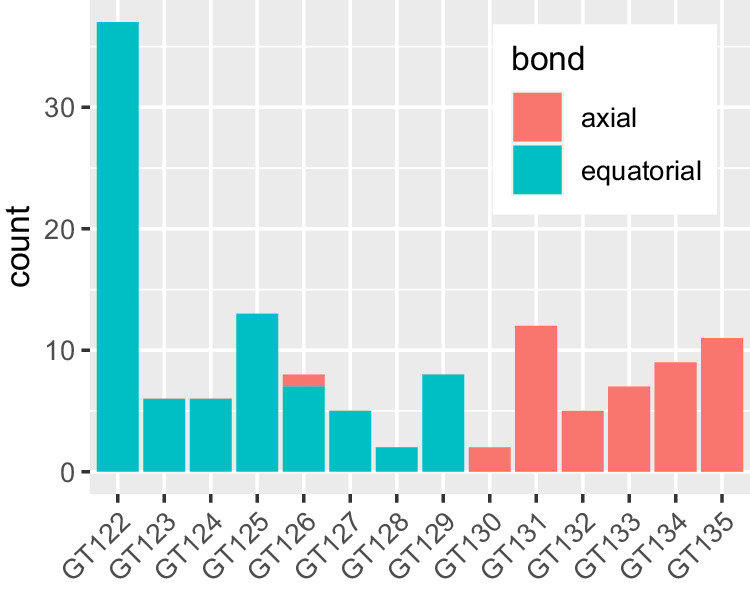
Level of conservation of stereochemical outcome of the reaction catalyzed in the various BP-Pol families. The bars represent the number of enzymes that are known to employ either retaining (making an axial bond) or inverting (making an equatorial bond) mechanisms in each of the new BP-Pol families.

We found that the stereochemical outcome of BP-Pols appears well conserved within the new BP-Pol CAZy families and varies from one family to another (Fig. 2). There is only one exception; in family GT126, the polymerase linkages are all equatorial except for the O-antigen in *Pseudomonas aeruginosa* O4, where it is axial. This could be due to an error in the chemical structure or the serotype designation or that the *P. aeruginosa* O4 polymerase constitutes an exception.

**Fig. 3:**
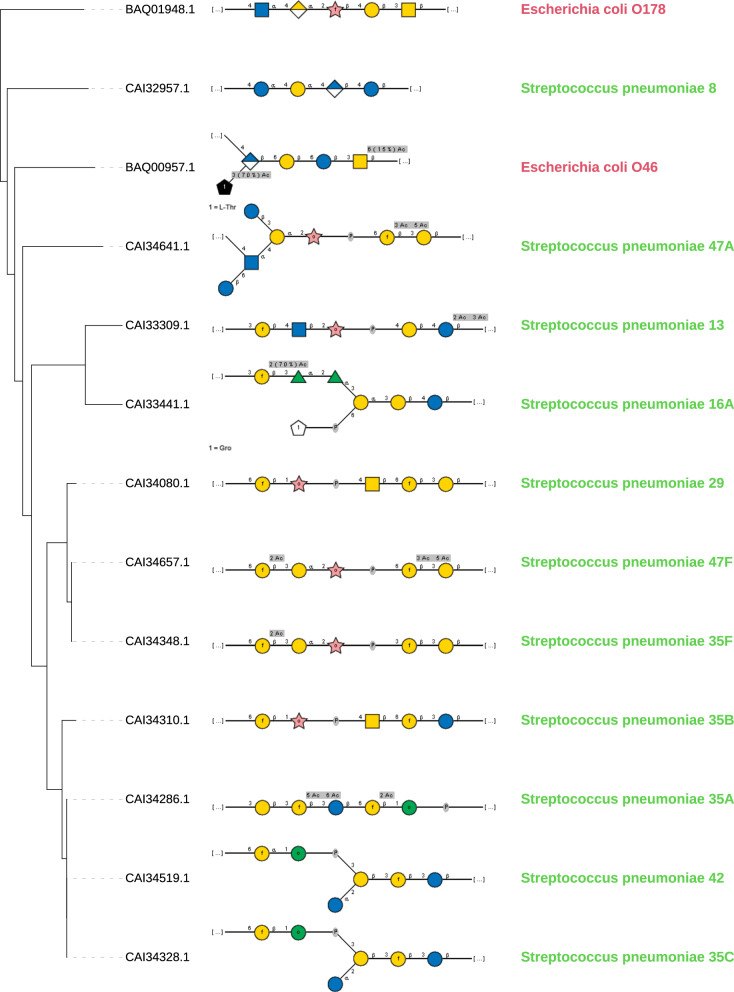
Comparison of repeat unit sugars transferred by BP-Pols in GT125. The transferred repeat unit structures (in SNFG representation) are shown on a phylogenetic tree of BP-Pols in family GT125. There is an overall similarity between all the transferred sugars in the family and the similarity appears to correlate with the tree structure, i.e., BP-Pol sequence similarity. In particular, the ends of the repeat units (+1 and -1 subsites) appear to be often conserved, whereas there is more variety in the central region where the enzyme does not interact with the sugar. Note that the +1 site corresponds to the non-reducing end of the depicted sugar structures and the -1 site corresponds to the reducing end. Notably, the family contains BP-Pols from distant taxonomic origin and that yet transfer similar repeat units.

**Fig. 4:**
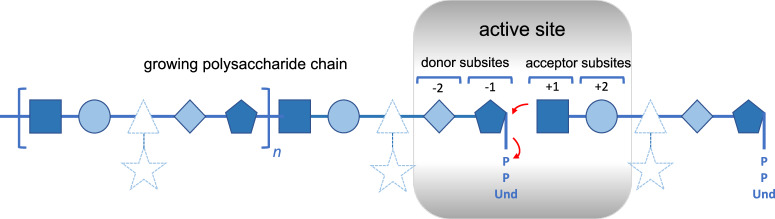
An idealized representation of a BP-Pol. The donor is the growing glycan chain activated by Und-PP while the acceptor is a single repeat unit linked to Und-PP. The reaction is hypothesized to chiefly involve the sugar residues of the donor (subsites −2 and −1) and of the acceptor (subsites +1 and +2) that are proximal to the reaction center rather than residues and branches that are more distal (depicted in dashed lines). The reaction is represented by red arrows.

Next, we investigated whether there was a correlation between the structures of the transferred sugars and the sequence similarity of the BP-Pols. We created phylogenetic trees of the BP-Pols in each family and visualized them with the corresponding transferred repeat units. We observe that the sugars within each family show similarity and this similarity appears to correlate with the structure of the tree, implying that polymerases with similar sequence utilize similar substrates (Fig. 3, Supplementary Fig. 4). The ends of the repeat units, ie. the subsite moieties immediately upstream (+1) and downstream (-1) of the newly created bond (Fig. 4) seem to be most conserved whereas more variability occurs in the middle part. We hypothesize that the +1 and -1 subsites are the moieties most important for recognition by the active site of the BP-Pol.

We observe examples of BP-Pols from distant taxonomic origin that cluster in the same CAZy family and have highly similar sugars. For example, *Escherichia coli* O178 and *Streptococcus pneumoniae* 47A in GT125 transfer sugars with almost identical backbones, suggestive of horizontal gene transfer (Fig. 3). There is only a slight variance in the middle of the repeat unit. This suggests that there is less constraints on the central part of the repeat unit than on the extremities that define the donor and the acceptor.

We next attempted to quantify the correlation between BP-Pol sequence and carbohydrate structure. For this we developed an original pairwise oligosaccharide similarity score. In our scoring scheme, the similarity of two glycans is estimated by examining the −1 and +1 subsites, as we expect that these are the moieties most fitting the active site of the BP-Pol (Fig. 4). The minimum match between two oligosaccharides corresponds to identical moieties at both subsites −1 and +1, which yields a score of 2. Thereafter, the score increases by one unit for each additional match at contiguous subsites, −2, −3, etc., and +2, +3, etc., up to a maximum value of 7 subsites found for the glycans encountered in this study (for details see Methods).

**Fig. 5:**
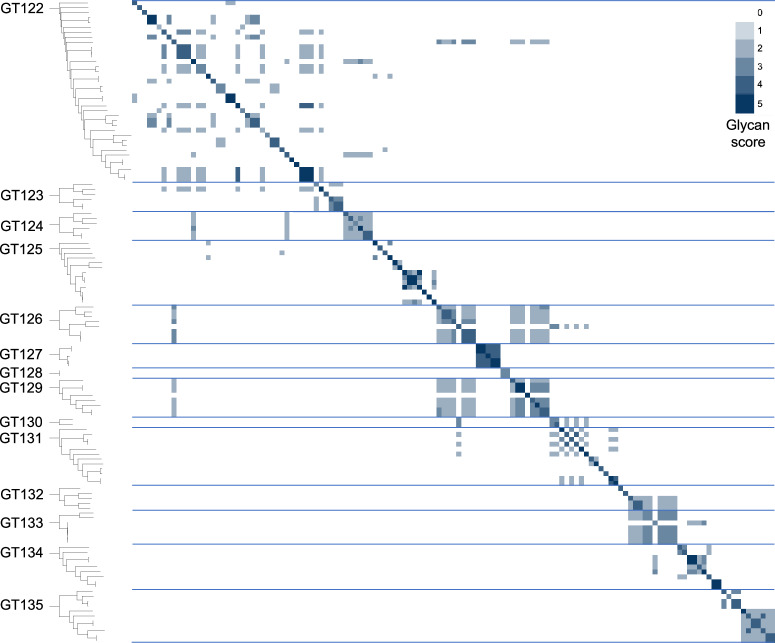
Glycan similarity of sugar repeat units polymerized by BP-Pols. All “seed” BP-Pols where the corresponding transferred oligosaccharide was known were included in the heatmap. A phylogenetic tree is shown for the polymerases in each CAZy family on the left. The glycan similarity scores are shown in a color scale of light blue (score value of 2 corresponding to identical matches at both −1 and +1 sites) to dark blue (score value of 5 corresponding to identical matches for at least three additional sequential positions). Horizontal lines separate the families. The darker colors close to the diagonal and within the families indicate specific substrate similarities in each family.

Using our glycan similarity scoring system, we found a correlation between sugar similarity and polymerase sequence similarity (Fig. 5), supported by a preponderance of similarity scores appearing close to the score matrix diagonal and within each individual family.

### Comparison of families

**Fig. 6:**
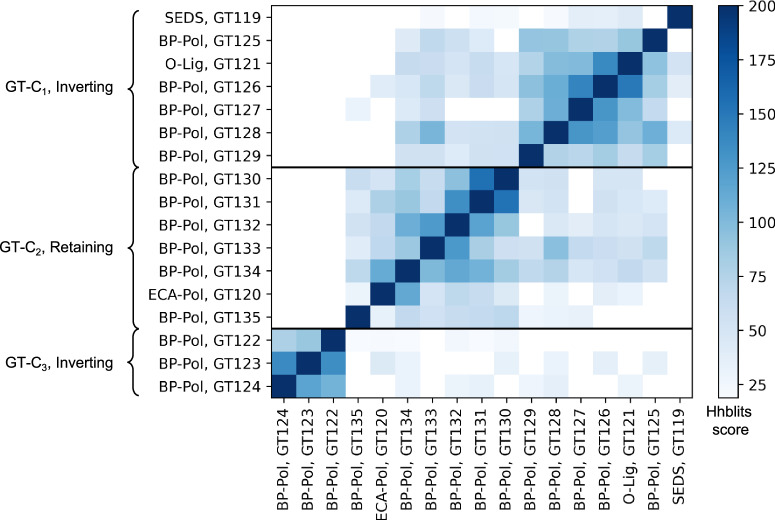
Relatedness of the new CAZy families and definition of clans. Inter-family HHblits bit scores are shown in a heatmap on a color scale from white (low similarity score) to dark blue (high similarity score). The HHblits scores depend on the direction of the alignment, and therefore the heatmap is not symmetrical. The inverting BP-Pols form two clans, GT-C_1_ which also contains the inverting SEDS (GT119) and the inverting O-Ligs (GT121) and GT-C_3_ containing only BP-Pols. The retaining BP-Pol families form one clan, GT-C_2_, which also contains the retaining ECA-Pol family (GT120).

Others have previously reported sequence and structural similarity between SEDS, O-Lig and some BP-Pols^13,14,21,23^. In order to investigate the relatedness of the new CAZy families, we compared the family HMMs by all-vs-all HHblits analyses^33^ (Fig. 6). Strikingly, we observe that the retaining BP-Pol families cluster together on the heatmap along with the retaining ECA-Pols, while the inverting BP-Pols form two distinct groups, one of them containing the inverting SEDS (GT119) and the inverting O-Ligs (GT121). The background noise between some inverting and retaining enzymes is likely due to the general conservation of the successive transmembrane helices, which is altered in the GT122-GT123-GT124 subgroup due to their different architecture (see below).

In the CAZy database, clans have been defined for the glycoside hydrolases (GHs), which group together CAZy families with distant sequence similarity, similar fold, similar catalytic machinery and stereochemical outcome^40^. In extension of the report of the GT-C_B_ class by Alexander and Locher^23^, and based on the above-mentioned similarities between the new CAZy families, we can now define three sequence-based clans: GT-C_1_ consisting of inverting BP-Pol families, SEDS and O-Lig, GT-C_2_ consisting of retaining BP-Pol families and ECA-Pol, and GT-C_3_ consisting of inverting BP-Pol families (Table 1, Fig. 6). The families within each clan share residual, local, sequence similarity, insufficient to produce a multiple sequence alignment, but suggestive of common ancestry. In the absence of a three-dimensional structure, and based on the sequence similarity to SEDS and O-Ligs, we have assigned the BP-Pol families of clan GT-C_1_ to the structural subclass GT-C_B_ of Alexander and Locher^23^. In addition, we also present in Table 1 the families of GT-C glycosyltransferases that have not yet been assigned to a structural class.

**Table 1:** Structural subclasses, clans and families of GT-C fold glycosyltransferases and relationships to mechanism and glycosyl donor

Structural subclass Alexander & Locher	CAZy clan	CAZy families	Mechanism	Donor
GT-C_A_	-	GT53	Inverting	Lipid-P-monosaccharide
GT-C_A_	-	GT83	Inverting	Lipid-P-monosaccharide
GT-C_A_	-	GT39	Inverting	Lipid-P-monosaccharide
GT-C_A_	-	GT57	Inverting	Lipid-P-monosaccharide
GT-C_A_	-	GT66	Inverting	Lipid-PP-oligosaccharide
GT-C_B_	GT-C_1_	GT119, GT121, GT125, GT126, GT127, GT128, GT129	Inverting	Lipid-PP-oligosaccharide
-	GT-C_2_	GT120, GT130, GT131, GT132, GT133, GT134, GT135	Retaining	Lipid-PP-oligosaccharide
-	GT-C_3_	GT122, GT123, GT124	Inverting	Lipid-PP-oligosaccharide
-	-	GT22	Inverting	Lipid-P-monosaccharide
-	-	GT50	Inverting	Lipid-P-monosaccharide
-	-	GT58	Inverting	Lipid-P-monosaccharide
-	-	GT59	Inverting	Lipid-P-monosaccharide

**Fig. 7:**
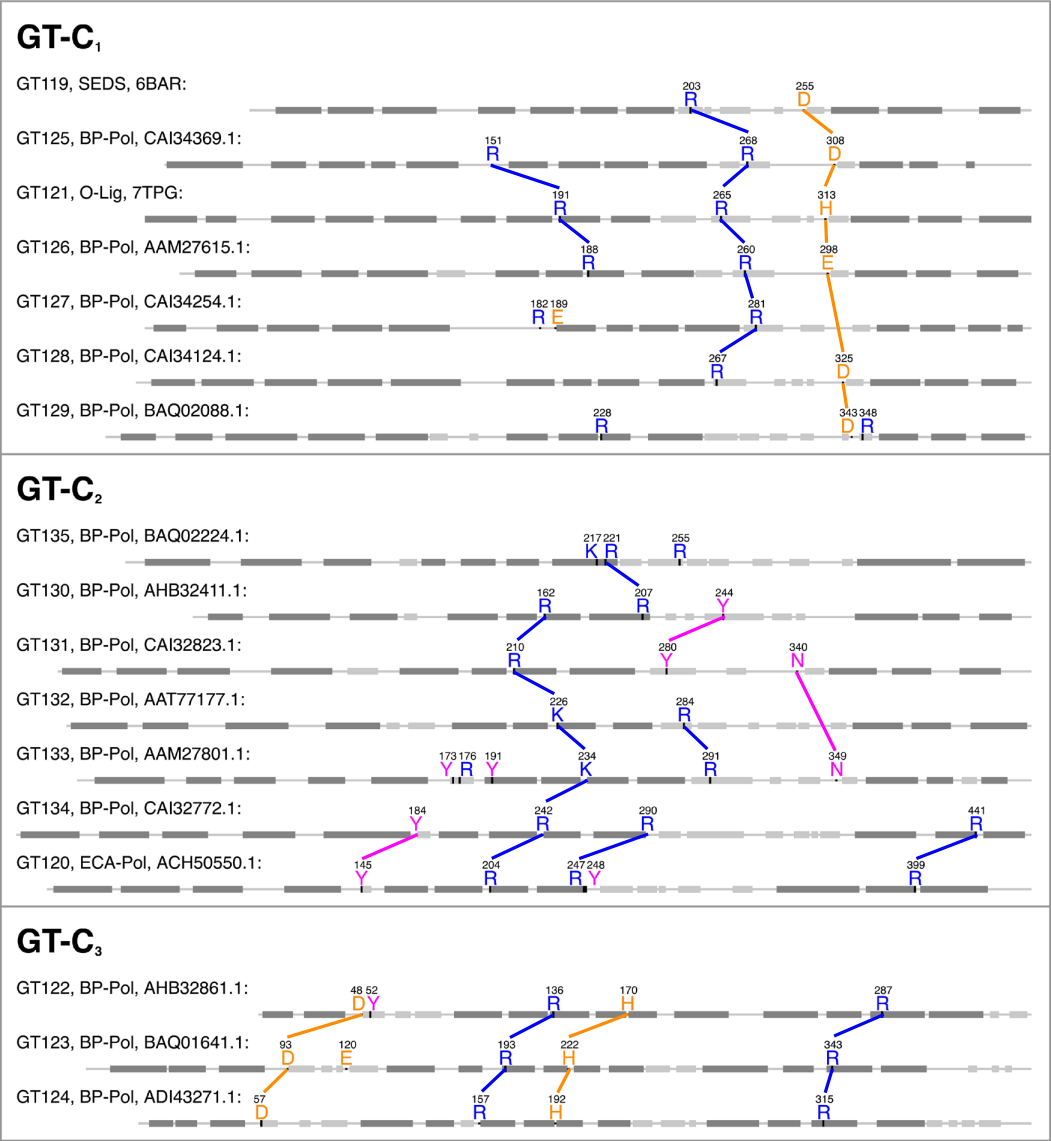
Equivalent conserved residues in the clans. Conserved residues of each of the new CAZy families are shown on sequences of representative family members. Colored lines are shown between conserved residues from different families, which align in HHblits alignments and co-localize in structural superimpositions (Supplementary Figs. 5–7). Transmembrane helices are shown in dark gray boxes, extracellular helices are shown in light gray boxes. The secondary structures were taken from the crystal structures for family GT119 and GT121 (6BAR and 7TPG respectively) and from AlphaFold models for all other families. The R210 in GT131 is either K or R in the family.

We then examined residue conservation and the general architecture of the enzymes in the clans. Based on the above mentioned pairwise HHblits analyses and structural superimpositions (Supplementary Figs. 5–7), we tried to evaluate which architectural features and conserved residues are common within the clans. Indeed, there are some common features across most families. In all the families, all the conserved residues are located on the outer face of the membrane (Fig. 7). Enzymes of clans GT-C_1_ and GT-C_2_ have a long extracellular loop close to the C-terminus containing conserved residues (Fig. 7). In stark contrast, families GT122, GT123 and GT124 of clan GT-C_3_ have an architecture completely different from that of the two other clans (Fig. 7), with a long loop located close to the N-terminus.

**Fig. 8:**
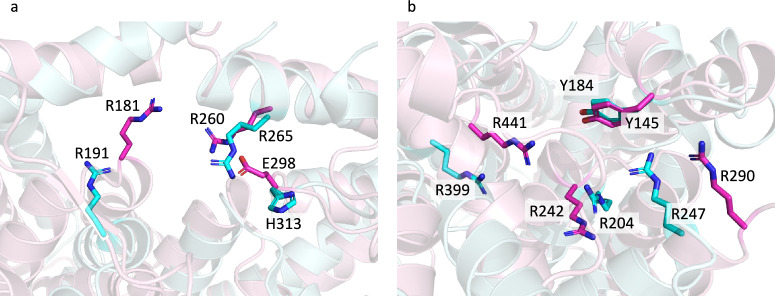
Structural superimpositions of members of different functional classes belonging to the same clans. **a** Superimposition of O-Lig in cyan (GT121, PDB: 7TPG) and AlphaFold model of BP-Pol in pink (GT126, Genbank accession: AAM27615.1) showing that the conserved Glu in the BP-Pol aligns with the conserved His in the O-Lig, which has been proposed to activate the acceptor^21^ (RMSD 5.3 Å over 192 residues, sequence identity 20.8% over 485 residues). **b** Structural superimpositions of AlphaFold models of ECA-Pol in cyan (GT120, Genbank accession: ACH50550.1) and BP-Pol in pink (GT134, Genbank accession: CAI32772.1) illustrating structural similarity and co-localization of the conserved residues (RMSD 5.4 Å over 360 residues, sequence identity 17.1% over 543 residue).

The families in GT-C_1_ show a distinct pattern of residue conservation. As mentioned above, the structure of O-Lig in complex with Und-PP revealed several important residues; Arg-191 and Arg-265 which bind to the phosphate groups of Und-PP, and His-313 which is proposed to activate the acceptor^21^. The SEDS family (GT119) also has a conserved Arg which aligns with the second conserved Arg in O-Lig and a conserved essential Asp which aligns with the conserved His in O-Lig (Fig. 7). Likewise, all the BP-Pols in the clan have 1–2 conserved Args, some of which align to the O-Lig Args in the HHblits alignments, and we hypothesize that they also play the role of binding to the diphosphate. Similarly, all the families in the clan except for GT127 have either a conserved Asp or Glu, which align with the conserved His of O-Lig and the conserved Asp of SEDS (Fig. 7). We hypothesize that these Glu and Asp residues also play the role of activating the acceptor. As an example, the superimposition of the published O-Lig structure (7TPG)^21^ and an AlphaFold model from one representative of the inverting BP-Pol family GT126 is shown in Fig. 8a. The superimposition produced an overall RMSD of 5.3 Å over 192 residues. Even with such a high RMSD, the two conserved Args are oriented very similarly, and the conserved His and Glu are in the same position. As mentioned above, GT127 does not have a conserved Asp or Glu in the same position as the rest of the families. However, it has a conserved Glu in a loop between transmembrane helices 5 and 6, which likely plays the same role.

In the retaining clan GT-C_2_, the pattern of conservation is different. Here, most of the families have 2–3 conserved Arg/Lys and 1–2 conserved Tyr (Fig. 7). Interestingly, we observe that the ECA-Pol family GT120 shows high similarity with one of the BP-Pol families, GT134. A superimposition of AlphaFold models from each family shows that the conserved residues are oriented very similarly, despite the low overall similarity (RMSD 5.4 Å over 360 residues) (Fig. 8b).

The families in the inverting clan GT-C_3_ all have two conserved Arg, a conserved Asp, and a conserved His, all of which align between the families in the HHblits alignments (Fig. 7).

## Discussion

Here we have added 17 glycosyltransferase families (GT119 to GT135) to the CAZy database bringing the total of covered families from 118 to 135. In the CAZy database, families are built by aggregating similar sequences around a biochemically characterized member. The known difficulties in the direct experimental characterization of integral membrane GTs render this constraint impractical. To circumvent this problem, but to remain connected to actual biochemistry, we decided to build our families around seed sequences for which knowledge of the glycosidic bond formed could be deduced from examination of the polysaccharide product from the literature.

To our knowledge, this is the first time that BP-Pols from different species have been successfully clustered. Indeed, forming groups of BP-Pols has been very difficult previously because of their extreme diversity even within strains of a single species^28^, and, as a consequence, the knowledge on conserved and functional residues has been very limited. By combining BP-Pols from a wide range of taxonomical origins and expanding with the current sequence diversity, we were able to form larger families of similar polymerases from widely different taxonomies, thereby revealing conserved residues that are most likely functionally important.

Because families are more robust when built with enough sequence diversity, many clusters of O-antigen polymerases were judged too small to build meaningful CAZy families. Additional polymerase families are thus expected in the future with the accumulation of sequence data. For instance the small cluster that contains 47% identical BP-Pols from *E. coli* O108 (GenBank BAQ01516.1) and *A. baumanii* O24 (GenBank AHB32586.1) only contains eight sequences and will remain unclassified until enough sequence diversity has accumulated. This arbitrary decision comes from the need to devise a classification that can withstand a massive increase in the number of sequences without the need to constantly revise the content of the families.

Moreover, we observe that the sequence diversity within the families we have built is minimal for peptidoglycan polymerases (GT119) and ECA-Pols (GT120), and then increases gradually for O-Ligs (GT121) and is maximal for BP-Pols (GT122-GT135). We hypothesize that sequence diversity reflects the donor and acceptor diversity in each family since the latter increases accordingly; the enzymes in the SEDS and ECA-Pol families act with the same donor and same acceptor, the enzymes in the O-Lig family act with different donors but same acceptor, and for the enzymes in the BP-Pol families act on different donors and different acceptors.

It has been observed that for classical GT-A and GT-B fold glycosyltransferases, the catalytic mechanism is conserved within a family, but families with the same fold can have different mechanisms, possibly because the stereochemical outcome of the glycosyl transfer reaction is essentially dictated by the precise positioning and activation of the acceptor above (S_N_2) or below (S_N_i) the sugar ring of the donor^4^. Very occasionally, retaining glycosyltransferases have been shown to operate via a double displacement mechanism that involves Asp/Glu residues to form a glycosyl enzyme intermediate and to activate the acceptor that attacks this intermediate^41^. The families defined here display globally similar GT-C folds, and they also show conservation of the catalytic mechanism with about half of the families retaining and the other half inverting the anomeric configuration of the donor, suggesting that the outcome of the reaction catalyzed by GT-C glycosyltransferases is also dictated by the positioning of the acceptor with respect to the sugar plane of the acceptor. In turn this also suggests that retaining BP-Pols also operate by an S_N_i mechanism rather than by the formation of a glycosyl enzyme intermediate. This hypothesis is supported by the lack of invariant Asp or Glu residues which could be involved in the formation and subsequent breakdown of a glycosyl enzyme intermediate in the retaining families GT120 and GT130-GT135. Additionally, the S_N_i mechanism may provide protection against the interception of a glycosyl enzyme intermediate by a water molecule resulting in an undesirable hydrolysis reaction and termination of the polysaccharide elongation.

The wealth of structural data of GT-C glycosyltransferases now permits a deeper evaluation of the intrinsic properties of this large class of enzymes. Alexander and Locher have recently evaluated the structural similarities between GT-C fold glycosyltransferases and have divided them in two fold subclasses^23^. The GT families that we describe here significantly expand the GT-C class in the CAZy database (www.cazy.org) and allow to combine the structural classes with mechanistic information. Lairson et al. have proposed the subdivision of GT-A and GT-B fold glycosyltransferases in clans that integrate the stereochemical outcome of the reaction^4^. Here we also note the conservation of the stereochemistry in the families of BP-Pols and we thus propose to group them into three clans which share the same fold, residual sequence conservation and the same catalytic mechanism (Table 1). As more families of BP-Pols emerge, these three clans will likely grow. Table 1 shows the three clans we defined here and how they relate to the structural classes defined by Alexander and Locher. Of note are families GT122, GT123, and GT124 which do not bear any similarity, even distant, with the GT families of the other two clans. These three families also stand out by the location in the sequence of the long loop that harbors the catalytic site in the other GT-C families. In absence of relics of sequence relatedness to the other families, GT122, GT123 and GT124 were assigned to clan GT-C_3_.

The analysis presented here shows that not only the stereochemistry of the glycosyl transfer is conserved in the BP-Pol families, but our development of an original method to estimate glycan similarity also reveals a certain degree of structural similarity of the oligosaccharide repeat units, suggesting that the latter constitutes a significant evolutionary constraint applying to the sequence and structure of BP-Pols. A closer inspection of the oligosaccharide repeat units within the families further reveals that the carbohydrates that appear the most constrained are the carbohydrates located (i) at the non-reducing end of the acceptor and (ii) close to the Und-PP of the donor, i.e. the residues closest to the reaction center (Fig. 4). By contrast, residues away from the two extremities engaged in the polymerization reaction appear more variable, and can tolerate insertions/deletions or the presence of flexible residues such as linear glycerol or ribitol, with or without the presence of a phosphodiester bond.

The version of the glycan similarity score presented here was inspired in part by observed structural similarities in different O-antigen repeat units assembled by very similar BP-Pols^16^. The repeat unit comparison involves a translation of glycan IUPAC nomenclature to a reduced alphabet of terms representing only backbone configuration, i.e., ignoring chemical modifications and sidechains. Furthermore, a positive similarity score requires an entire identical match of all backbone elements at both donor and acceptor positions (−1 and +1 sites in Fig. 4, respectively). Despite these simplifications, the similarity score reveals, with exceptions, an overall greater intra- rather than inter-family oligosaccharide similarity (Fig. 5). These limitations will be addressed at a later stage (G.P. Gippert, in preparation).

We have next looked at the distribution of the new GT families in genomes, and particularly the families of BP-Pols. This uncovers broadly different schemes, with some bacteria having only one polymerase (and therefore only able to produce a single polysaccharide) while others having several, and sometimes more than 5, an observation in agreement with the report that *Bacteroides fragilis* produces no less than 8 different polysaccharides from distinct genomic loci^42^. The multiplicity of polysaccharide biosynthesis loci in some genomes makes it sometimes difficult to assign a particular polysaccharide structure to a particular biosynthesis operon.

We observed that the O-Lig family (GT121) was present in many Gram-positive bacteria such as *Streptococcus pneumoniae*. The covalent anchoring of CPS in Gram-negative bacteria is still poorly understood, although it is found to be linked to peptidoglycan in some Gram-positive bacteria^17,43^. Thus a hypothesis could be that the GT121 members in *S. pneumoniae* are responsible for the ligation of CPS to the peptidoglycan layer in these bacteria.

As already shown in other occasions, the sequence-based classification of carbohydrate-active enzymes of the CAZy database has predictive power. The case of the GT families described here supports this view as the invariant residues in the families not only co-localize in the same area of the three-dimensional structures (whether actual or AlphaFold-predicted), but also correspond to the residues found essential for function in the families where this has been studied experimentally. The families described herein also show mechanistic conservation and thus the stereochemistry of glycosyl transfer can be predicted. Finally, the observed similarity in oligosaccharide repeat units that accompanies sequence similarity has also predictive power and paves the way to the future possibility of in silico serotyping based on DNA sequence.

## Methods

### Alignment-based Clustering (Aclust)

Phylogenetic trees were generated using an in-house tool called Aclust (G.P.Gippert, manuscript in preparation). Aclust employs a hierarchical clustering algorithm comprising the following steps. (1) A distance matrix is computed from all-vs-all pairwise local sequence alignments^44^, or from a multiple sequence alignment provided by MAFFT^45^. The distance calculation is based on a variation of Scoredist^46^ where distance values are normalized to the shorter pairwise sequence length rather than to pairwise alignment length. (2) The distance matrix is embedded into orthogonal coordinates using metric matrix distance geometry^47^, and (3) a bifurcating tree is computed using nearest-neighbor joining and centroid averaging in the orthogonal coordinate space. The last centroid created in this process is defined as the root node. (4) Beginning with the root node of the initial tree, each left and right subtree constitutes disjoint subsets of the original sequence pool, which are reembedded and rejoined separately (i.e., steps 2 and 3 repeated for each subset), and the process repeated recursively—having the effect of gradually reducing deleterious effects on tree topology arising from long distances between unrelated proteins.

### Building the peptidoglycan polymerase family (GT119)

The peptidoglycan polymerase family, GT119, was built by using Blastp from BLAST+ 2.12.0+^48^ with the sequences of the characterized SEDS proteins (PDB 6BAR, 8TJ3, 8BH1 and GenBank accession CAB15838.1) against GenBank with a threshold of approximately 30% to retrieve the family members. Next, an MSA was generated with MAFFT v7.508 using the L-INS-i strategy^45^, and an HMM model was built with hmmbuild of HMMER 3.3.2^49^. The family was further populated using hmmsearch from HMMER 3.2.2 against GenBank.

### Building the enterobacterial common antigen polymerase family (GT120)

A sequence library of ECA-Pols was constructed by using Blastp with the seed sequence (GenBank accession AAC76800.1) against the NCBI non-redundant database version 61 with an E-value threshold of 1e-60. The hits were redundancy reduced using CD-HIT 4.8.1^50^ with a threshold of 99%. The redundancy-reduced pool of ECA-Pol sequences was clustered using our in-house tool Aclust (see above), and the tree showed one large clade and a few outliers. All the sequences in the large clade were used to build an MSA using MAFFT v7.508 with the L-INS-i strategy^45^. An HMM was built based on this MSA using hmmbuild of HMMER 3.3.2^49^. The family GT121 was built in CAZy and populated using Blastp against GenBank with an approximate threshold of 30% and hmmsearch against GenBank.

### Building the O-antigen ligase family (GT121)

37 O-Lig sequences were selected from literature (Supplementary Data 1) and expanded using Blastp against the NCBI non-redundant database with an E-value cut-off of 1e-60. Redundancy reduction was performed on the resulting sequence pool using CD-HIT with a threshold of 99%, resulting in a pool of 1402 sequences. A phylogenetic tree of the pool of O-Lig sequences was generated using Aclust (see above), which showed deep clefts between main branches, and branches with sufficient internal diversity (Supplementary Fig. 2). Based on these results, four subfamilies were determined. An MSA was built for the family as well as for the subfamilies with MAFFT v7.508 using the L-INS-i strategy. HMMs were built based on the MSAs using the hmmbuild of HMMER 3.3.2^49^. The family was populated using Blastp against GenBank with an approximate threshold of 30% identity with the seed sequences and using hmmsearch with the family and subfamily HMMs.

### Building the bacterial polysaccharide polymerase families (GT122-GT135)

363 BP-Pol sequences were retrieved from review papers on biosynthesis of O-antigens and capsular polysaccharides in different species: *Escherichia coli*^28^, *Shigella boydii*, *Shigella dysenteriae*, *Shigella flexneri*^29^, *Salmonella enterica*^30^, *Yersinia pseudotuberculosis*, *Yersinia similis*^31^, *Pseudomonas aeruginosa*^16^, *Acinetobacter baumanii*, *Acinetobacter nosocomialis*^32^ and *Streptococcus pneumoniae*^19^ (complete list in Supplementary Data 2). The BP-Pols for *A. baumannii* O7 and O16 were omitted, because of uncertainty of their serotypes^32^. The BP-Pol from *P. aeruginosa* O15 was also omitted, because it has been shown that this BP-Pol is inactivated and that the O-antigen is synthesized via the ABC-dependent pathway rather than the Wzx/Wzy-dependent pathway^51^.

The sequence library was expanded using Blastp for each seed sequence against the NCBI non-redundant database with an E-value threshold of 1e-15. Redundancy reduction was performed using CD-HIT with a threshold of 95% identity.

To find clusters of BP-Pol sequences that were large enough to create a CAZy family, we developed a clustering method consisting of two steps. First, in order to make a sequence similarity network (SSN), all-vs-all pairwise local alignments of the BP-Pol sequence pool were performed using Blastp from BLAST+ 2.12.0+. A series of networks were built using different bit score thresholds. The members of the resulting SSN clusters were identified using NetworkX^52^ and MSAs of the members were built with MAFFT v7.508 using the L-INS-i strategy. The MSAs were inspected using Jalview^53^, and a bit score threshold of 110 was selected, as it was the lowest score for which the SSN clusters had adequate sequence conservation (approximately 15 conserved residues).

HMMs were then built for each SSN cluster using hmmbuild of HMMER 3.3.2, and the HMMs were compared using HHblits 3.3.0^54^. A series of HHblits networks were built using different HHblits score thresholds. Again, the members of the resulting superclusters were identified using NetworkX and MSAs of the superclusters were built with MAFFT v7.508 using the L-INS-i strategy. A bit score threshold of 160 was selected as it resulted in superclusters with adequate diversity for building CAZy families (approximately 5 conserved residues). CAZy families were created for the 14 largest superclusters and populated with sequences present in GenBank by a combination of Blastp with the seed sequences and hmmsearch. The networks were visualized with Cytoscape^55^.

### Analysis of sugar repeat unit structures

In order to analyze the relation between BP-Pol sequence and structure of the transferred repeat unit, we retrieved the repeat unit structures for the serotypes for the BP-Pols that were included in the new CAZy families. The repeat unit structures were retrieved from the same review papers from which we retrieved the BP-Pol sequences^16,19,29–32^, except for the sugars for *E. coli*, where the sugar structures have been reported elsewhere^34^. Nine additional repeat unit structures were included for *S. pneumoniae*, which were published after the review paper; serotypes 16A^35^, 33A^36^, 33C and 33D^37^, 35C and 35F^38^, 42 and 47F^56^ and 47A^57^. For *Y. pseudotuberculosis* O3 and *S. pneumoniae* 33B, we used the revised structures^37,39^. *Pseudomonas aeruginosa* O2 and O16 contain two BP-Pol genes; one BP-Pol localized in the O-antigen biosynthesis cluster, which polymerizes the sugar repeat units with an α bond and one BP-Pol localized outside the biosynthesis cluster which polymerizes the repeat units with a β bond^58^. Since the BP-Pols reported in^16^ are from the O-antigen cluster, we report the sugar structure with the α bond.

The linkages formed by the polymerase have been determined in all of these papers, except for a few cases. This determination is based on the other GTs in the gene cluster, in particular the initial GT which transfers the first monosaccharides to the Und-PP anchor. The cases where the polymerase linkage has not been unambiguously determined in the review papers are *E. coli* O166, O78, O152, O81, O83, O11, O112ab, O167, O187, O142, O117, O107, O185, O42, O28ac, O28ab, for which there are two or more possible polymerase linkages. For the structures that were published after the review papers, the polymerase bond had not been determined in *S. pneumoniae* 33A and 47A. For *S. pneumoniae* 33A, we determined the linkage based on the presence of the initial transferase *wchA* in the gene cluster, which transfers a glucose-1-phosphate to Und-PP^19^. In *S. pneumoniae* 47A the initial transferase is WcjG, which transfers Gal*p* or Gal*f* ^19^. Since the repeat unit contains both Gal and Galp, we could not determine the polymerase linkage unambiguously. However, the repeat unit is very similar to other repeat units in the family (most similar to that of *S. pneumoniae* 13), and we proposed the equivalent polymerase linkage.

The CSDB database (http://csdb.glycoscience.ru)^59^ was used to retrieve literature, SNFG image representations and linear sugar strings of the repeat unit structures. Phylogenetic trees for BP-Pol families with sugar structures were generated using MAFFT v7.508^45^ with the L-INS-i strategy to supply an initial multiple sequence alignment, followed by Aclust (see above) for distance matrix embedding and clustering. The trees were visualized in iTOL^60^. The barplot was generated using R^61^, Rstudio^62^, and the ggplot2 package^63^.

### Oligosaccharide backbone similarity score

A similarity score function was developed that quantifies the number of identical subunits at both donor and acceptor ends of oligosaccharides, specifically positions […, − 2, − 1, + 1, + 2, …] with respect to the bond formation site (Fig. 4). The minimum non-zero similarity score between a pair of oligosaccharides is 2, requiring identity at both positions -1 and +1. Thereafter the comparison extends by one position in each positive ( + 2, + 3, …) and negative (− 2, − 3, …) chain direction, adding one to the score for each additional identical match, but terminating at the first non-identity or possible re-use of a backbone position.

**Fig. 9:**
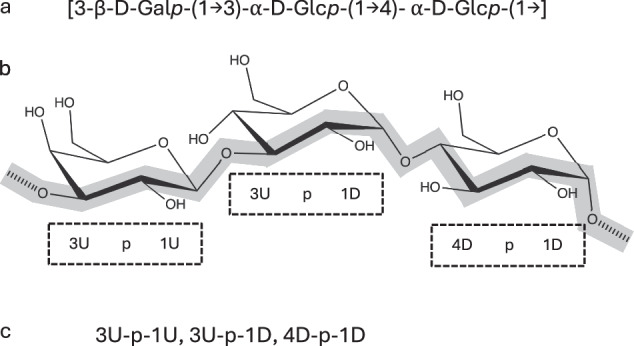
Oligosaccharide translation from IUPAC nomenclature to backbone (geometric) subunits for a trisaccharide consisting of one D-galactopyranose and two D-glucopyranose residues joined by intramolecular *β*1 → 3 and *α*1 → 4 bonds, respectively, and an intermolecular *α*1 → 3 bond formed in the polymerase reaction. **a** IUPAC nomenclature. **b** Stereochemical projection highlighting backbone (thick gray line) and transfer bond (hatched line segments), and translated geometric subunits below. **c** Completed translation.

To facilitate comparison, oligosaccharide sequences are translated from IUPAC nomenclature into symbols that represent elements of backbone geometry, only considering monomer dimension and stereochemistry of acceptor and anomeric donor carbon atoms, and ignoring sidechains and chemical modification (Fig. 9). Briefly, the monomer dimension is represented by a single letter P, F or L depending on whether the monomer sugar is a pyranose, furanose or is linear, respectively. Stereochemistry of the acceptor and donor carbon atoms is represented by the index number of the carbon position within the ring/monomer, followed by a single letter U, D or N depending on whether the linked oxygen atom is U (up=above the monomer ring), D (down=below the monomer ring), or N (neither above or below the ring). The N symbol is assigned in cases of conformational flexibility such as with alditols or C6 linkages. At present, in scoring the similarity of two thus translated residues, the entirety of the translation strings must be identical to achieve a score of +1. Further details and limitations will be presented elsewhere (G.P. Gippert, manuscript in preparation).

### Comparison of the families

Pairwise HHblits analyses^33^ were performed for each of the new CAZy families. The HHblits scores were visualized in a heatmap using Python Matplotlib^64^.

AlphaFold2^14^ structures were generated of representative proteins from the families using the ColabFold implementation^65^ on our internal GPU cluster processed with the recommended settings. The best ranked relaxed model was used. The protein structures were visualized in PyMOL^66^ and pairwise structural superimpositions were performed using the CEalign algorithm^67^.

### Reporting summary

Further information on research design is available in the Nature Portfolio Reporting Summary linked to this article.

### Supplementary information

Supplementary Figures

Description of Supplementary Materials

Supplementary Data 1

Supplementary Data 2

Reporting Summary

## Data Availability

Accessions to the seed sequences utilized in this work are given in Supplementary Data 1 and 2; the constantly updated content of families GT119 - GT135 is given in the online CAZy database at www.cazy.org.

## Code availability

Source code for Aclust may be obtained via GitHub at https://github.com/GarryGippert/Aclust.
