# Supplementary material for: Diversity of sugar-diphospholipid-utilizing glycosyltransferase families
Source: Commun Biol. 2024 Mar 7;7:285. doi: 10.1038/s42003-024-05930-2 (PMC10920833; doi:10.1038/s42003-024-05930-2)
Supplement: Supplementary file 1 — Supplementary Figures [file 42003_2024_5930_MOESM1_ESM.pdf]

Supplementary Information for

Diversity of sugar-diphospholipid-utilizing glycosyltransferase families

Ida K.S. Meitil, Garry P. Gippert, Kristian Barrett, Cameron J. Hunt, and Bernard Henrissat*.

*Corresponding author
E-mail: bernard.henrissat@gmail.com

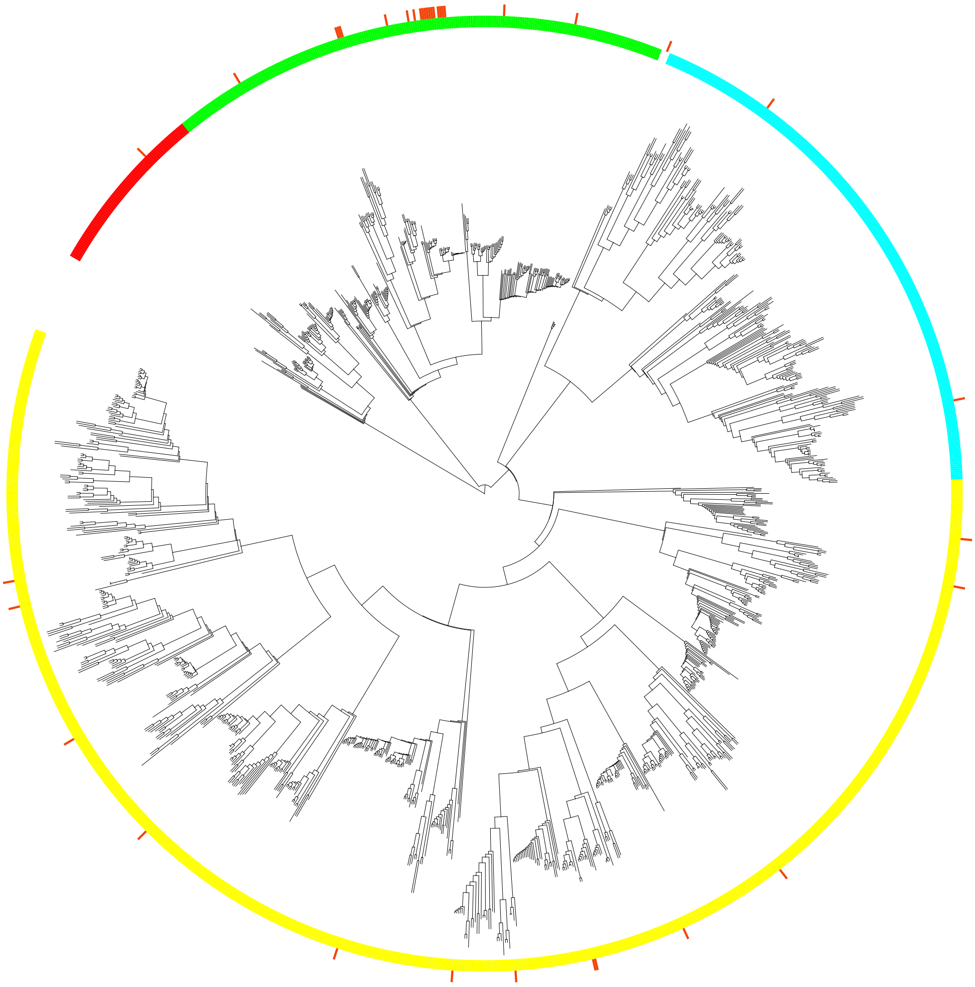

Supplementary Figure 1. Aclust tree of representative O-Lig sequences. The four subfamilies are shown in red, green, turquoise, and yellow. Seed sequences are marked with a red bar.

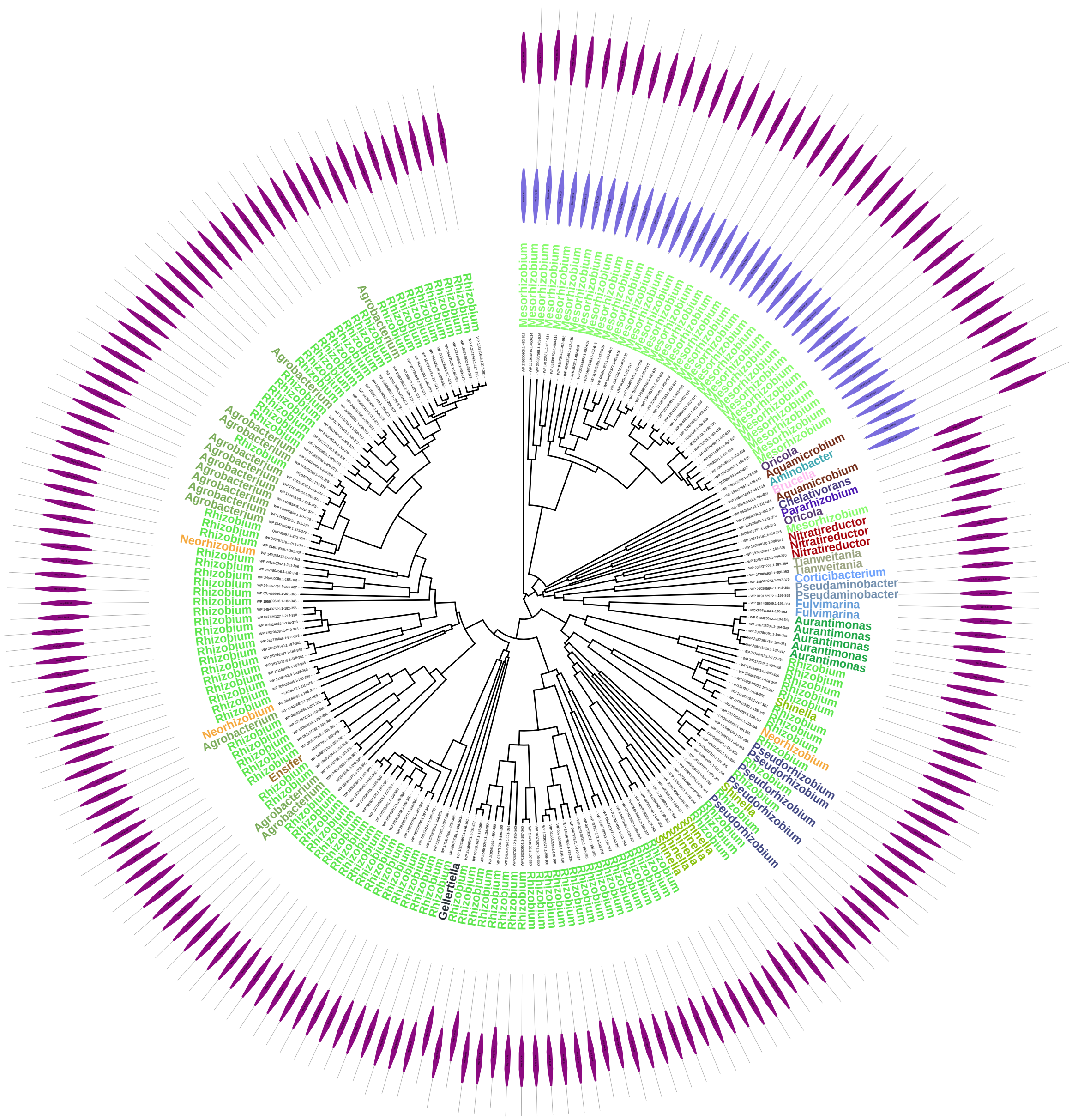

Supplementary Figure 2. Phylogenetic tree of representative WadA homologs from the GT121 family, showing the taxonomic origin of the sequences and the domain modularity. N-terminal GT25 domains are shown in blue and GT121 domain in purple. The two-domain sequences segregate from proteins with a single GT121 domain, suggesting co-evolution of the two domains.

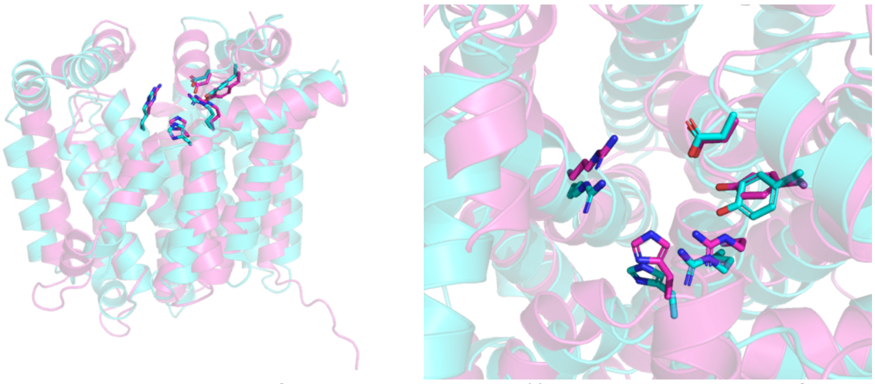

ABB29906.1 and AHB32334.1. Sequence identity: 21.4% over 421 residues. RMSD: 4.3Å over 304 residues.

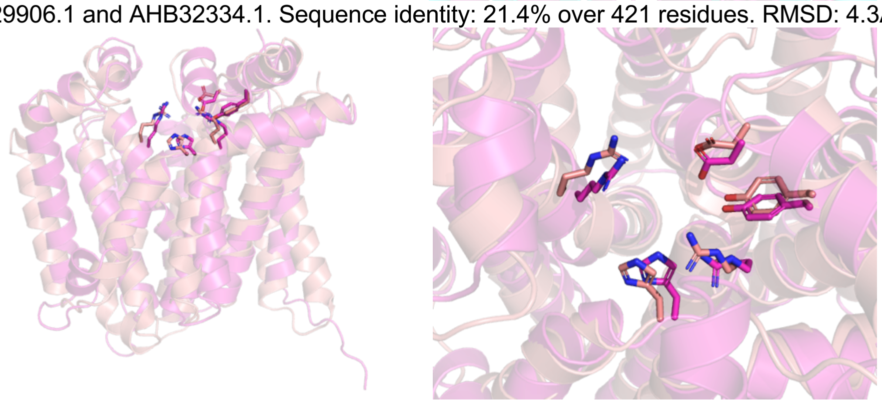

ACA24821.1 and AHB32334.1. Sequence identity: 21.7% over 415 residues. RMSD: 3.9Å over 312 residues.

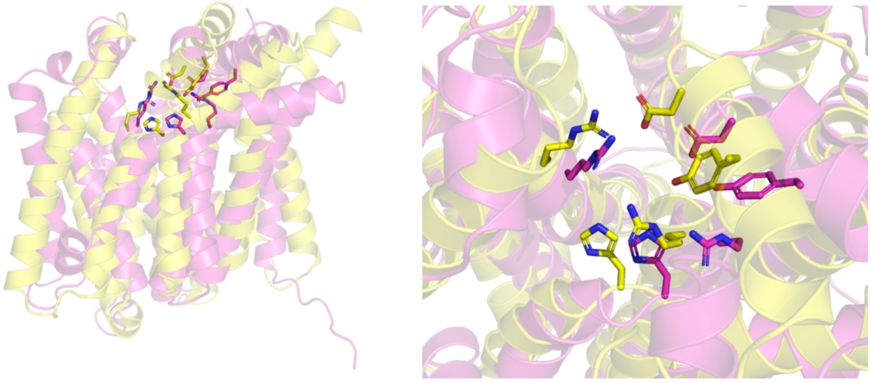

AHB32215.1 and AHB32334.1. Sequence identity: 24.3% over 403 residues. RMSD: 5.1Å over 248 residues.

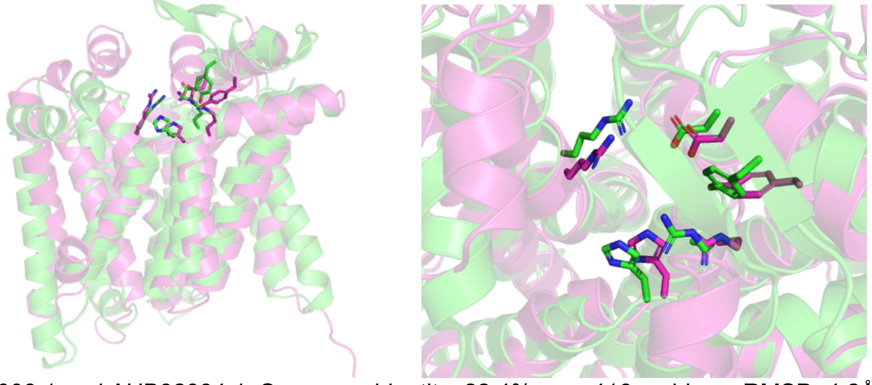

CAI34008.1 and AHB32334.1. Sequence identity: 22.4% over 416 residues. RMSD: 4.2Å over 320 residues.

Supplementary Figure 3. Structural superimpositions of AlphaFold models of distantly related BP-Pols from family GT122 aligned to a reference, AHB32334.1. AHB32334.1 is shown in pink in all of the figures.

GT122:

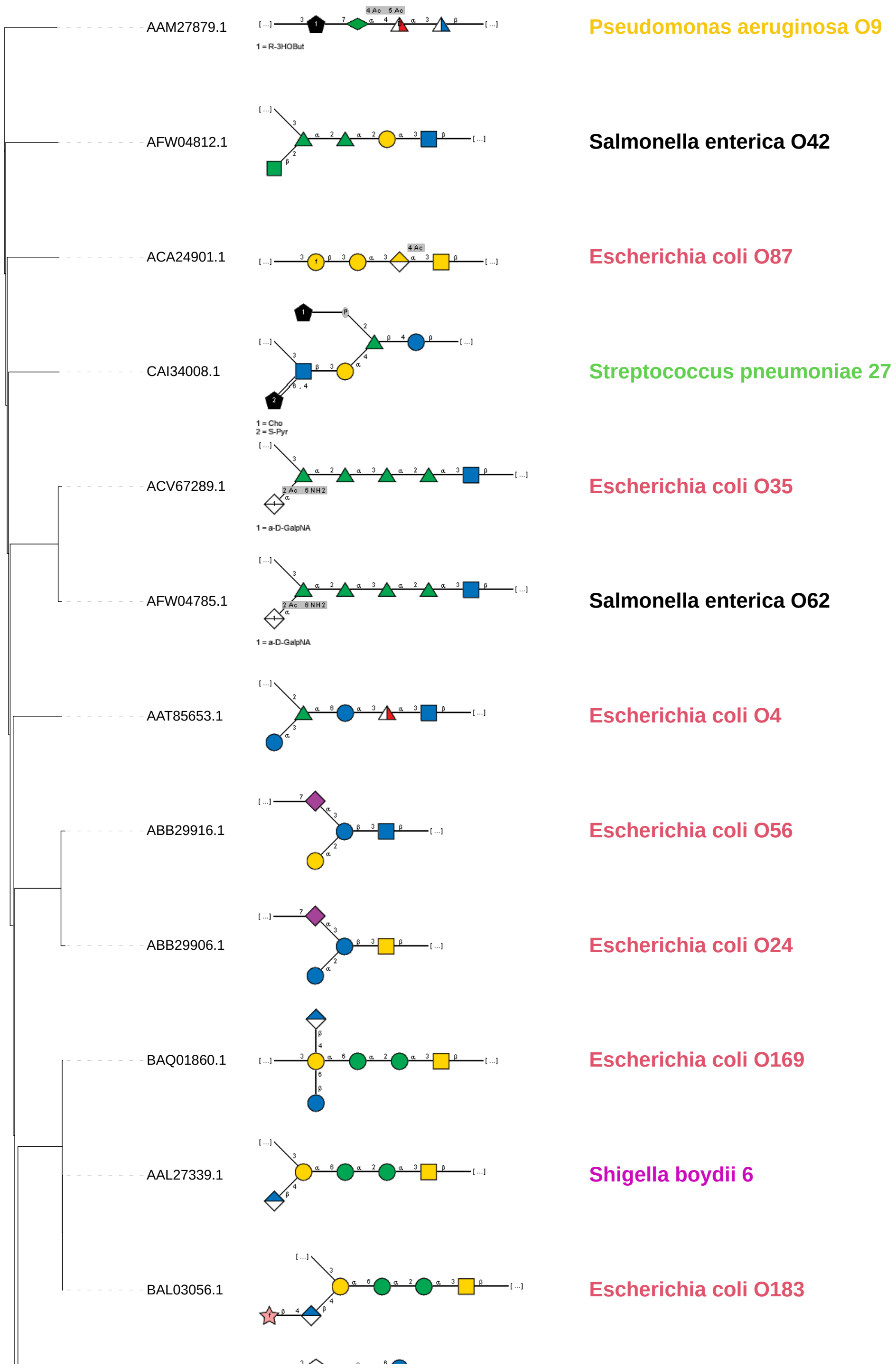

Supplementary Figure 4. Phylogenetic trees of the BP-Pols in each family with structures of the corresponding sugar repeat units in SNFG format visualized using iTOL. The tree for GT122 is split in three pages due to its size. No tree is shown for GT128, because the two BP-Pol members with known repeat units have identical sequences.

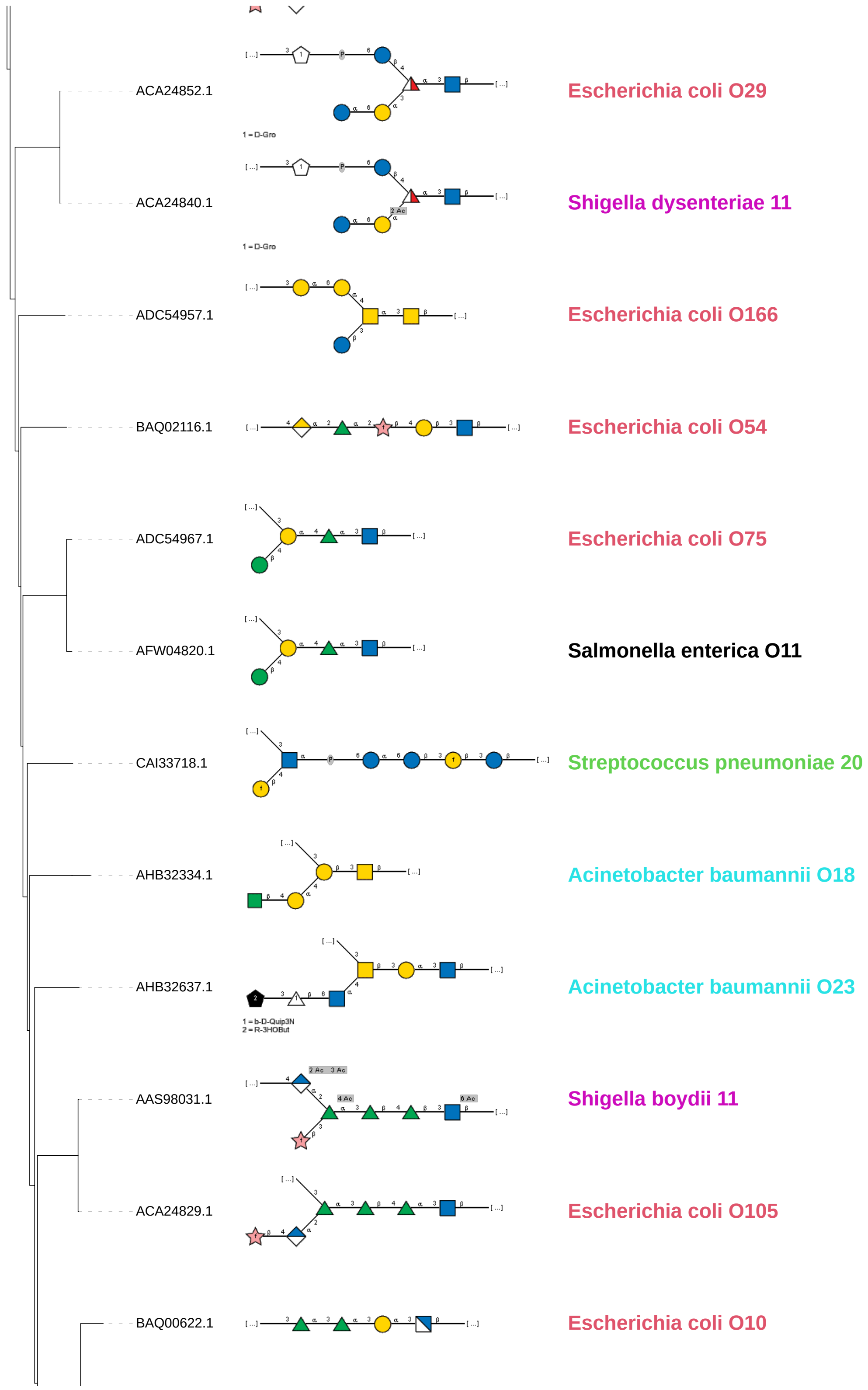

Supplementary Figure 4 – continued.

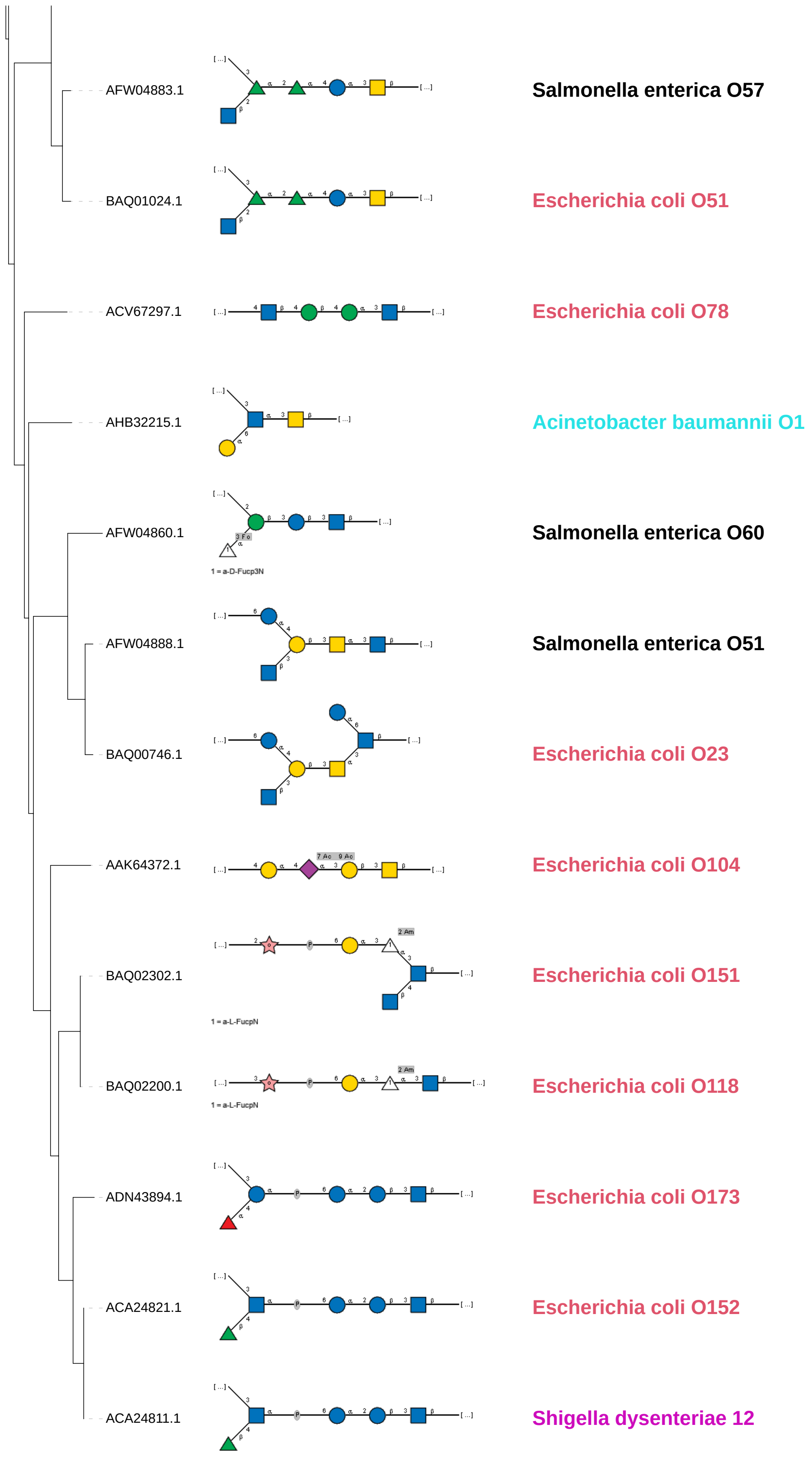

Supplementary Figure 4 – continued.

GT123:

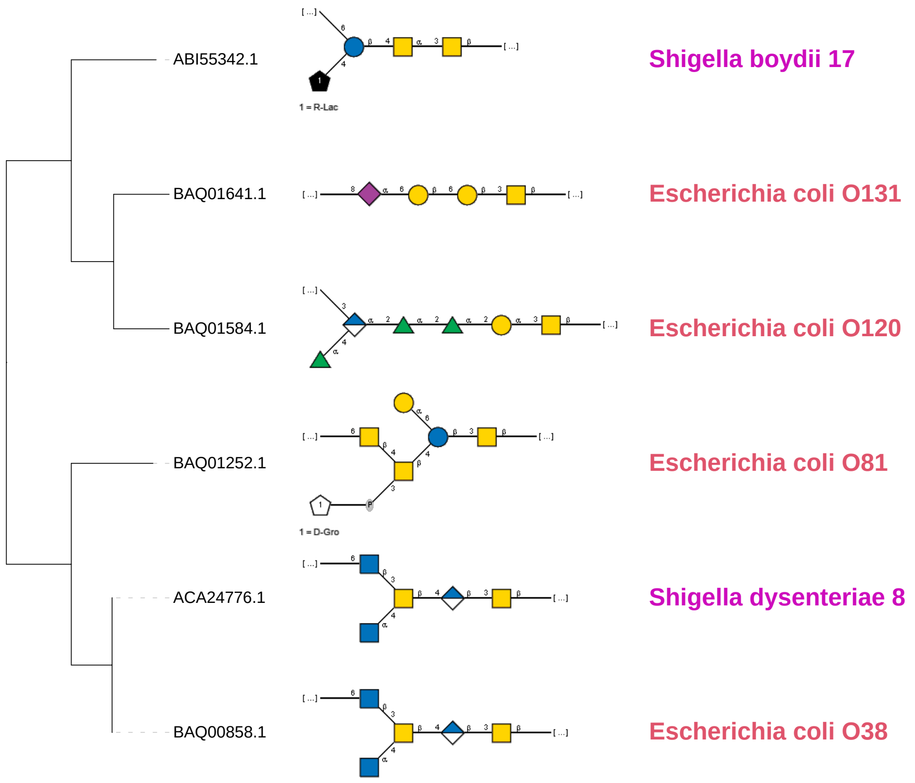

Supplementary Figure 4 – continued.

GT124:

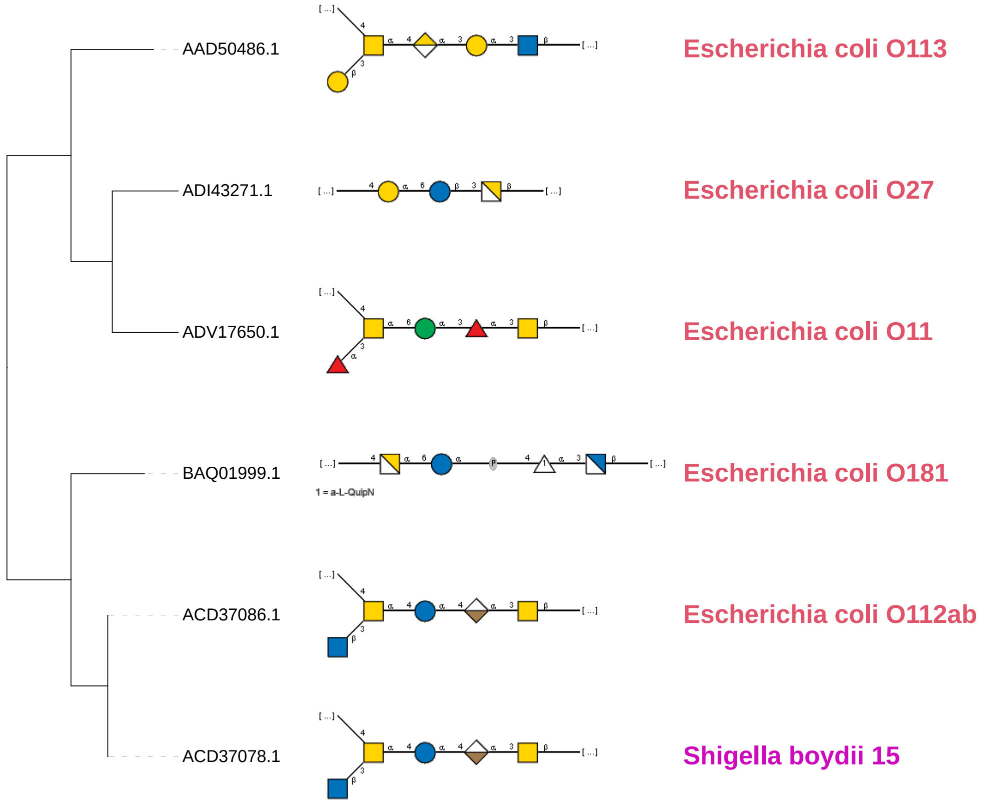

Supplementary Figure 4 – continued.

GT125:

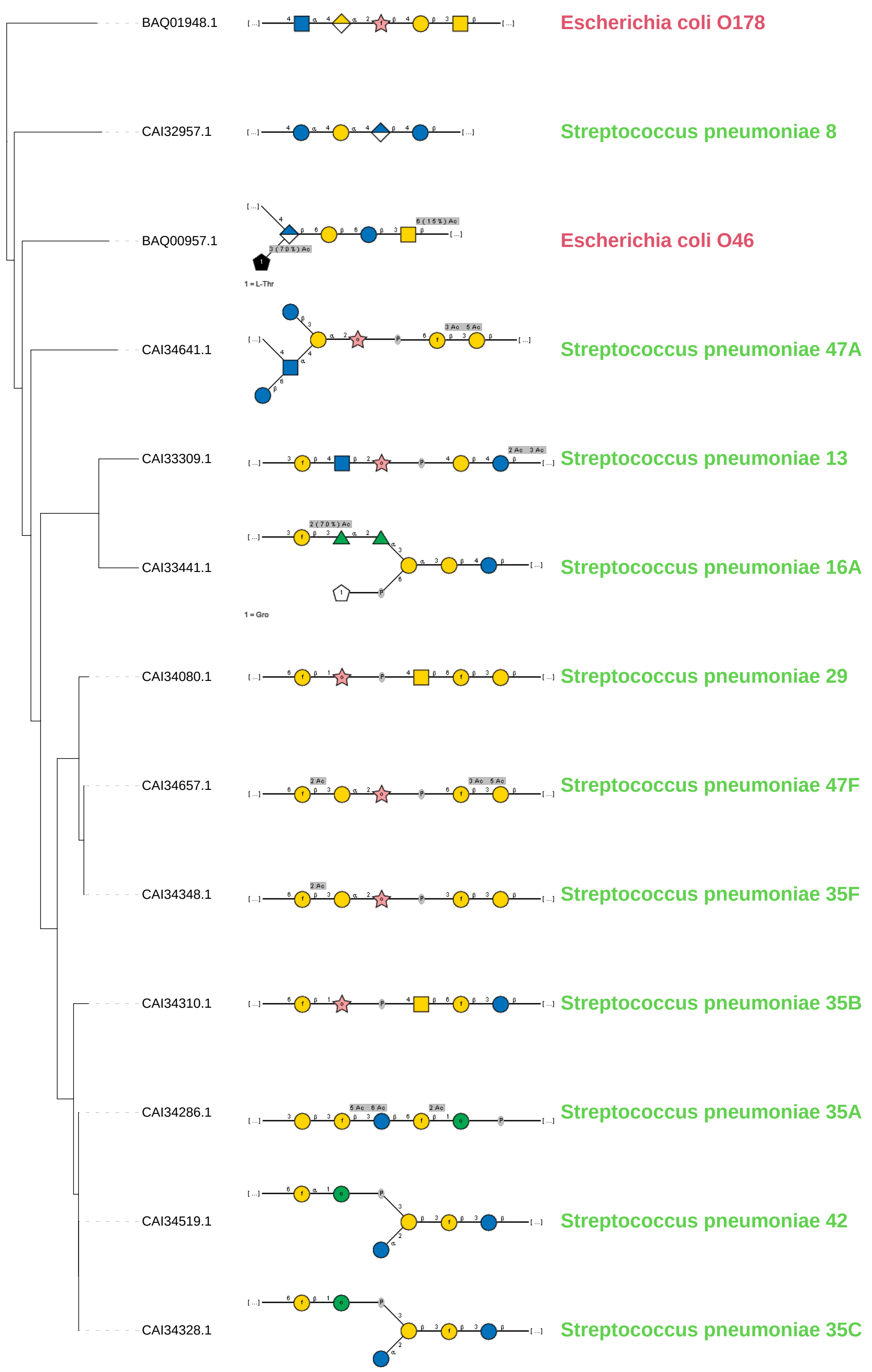

Supplementary Figure 4– continued.

GT126:

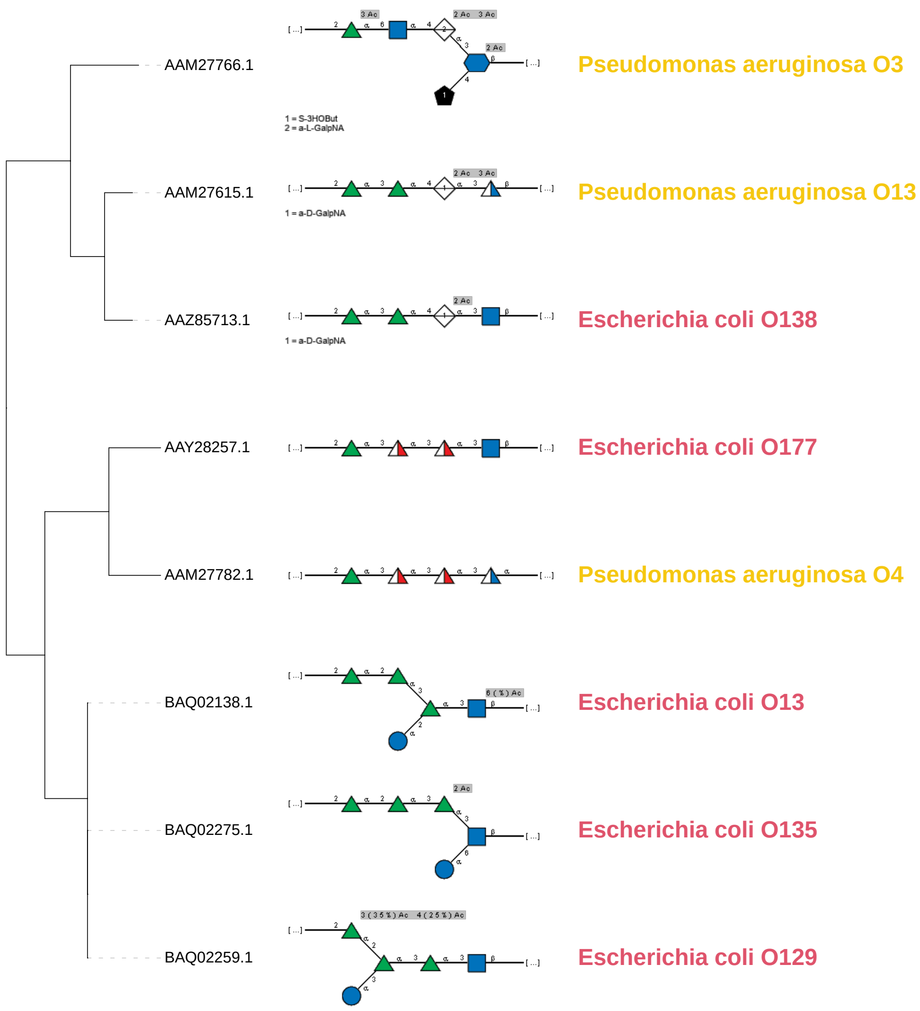

Supplementary Figure 4 – continued.

GT127:

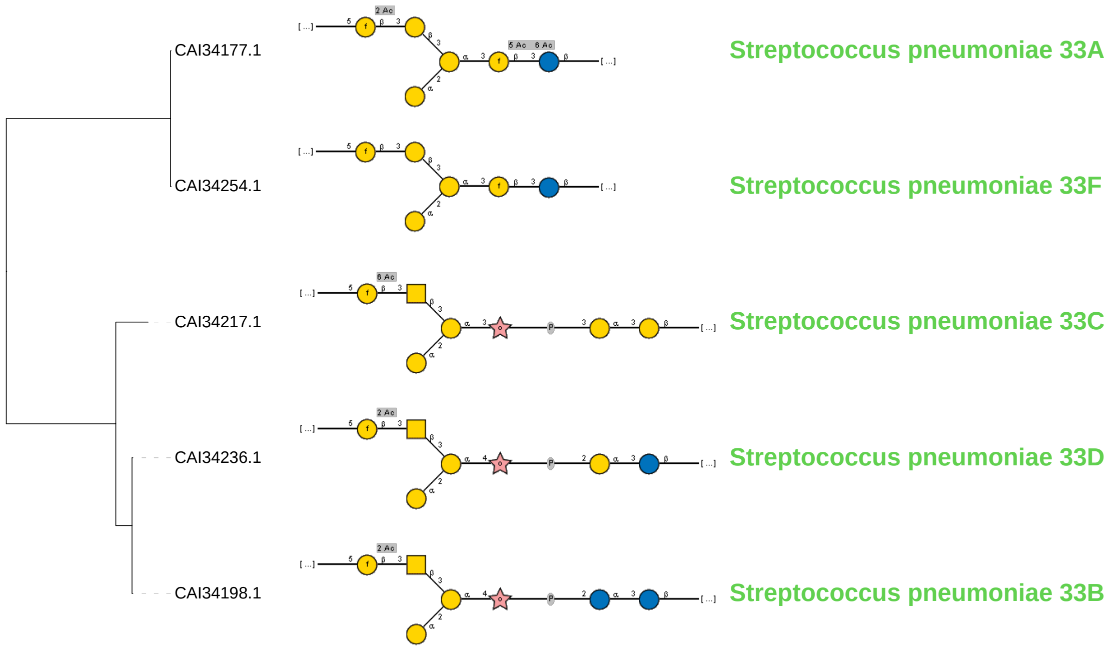

GT128:

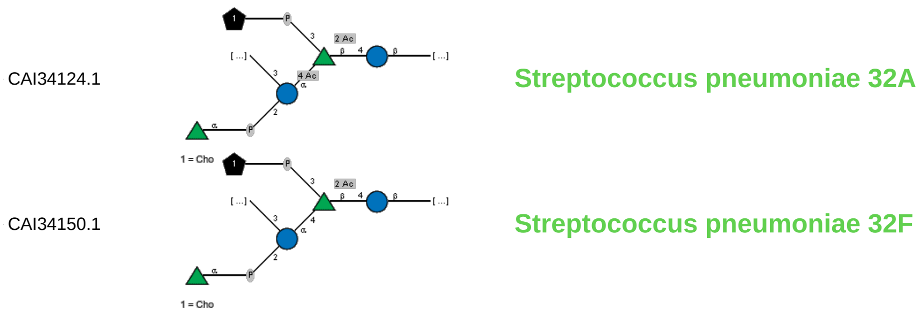

Supplementary Figure 4 – continued.

GT129:

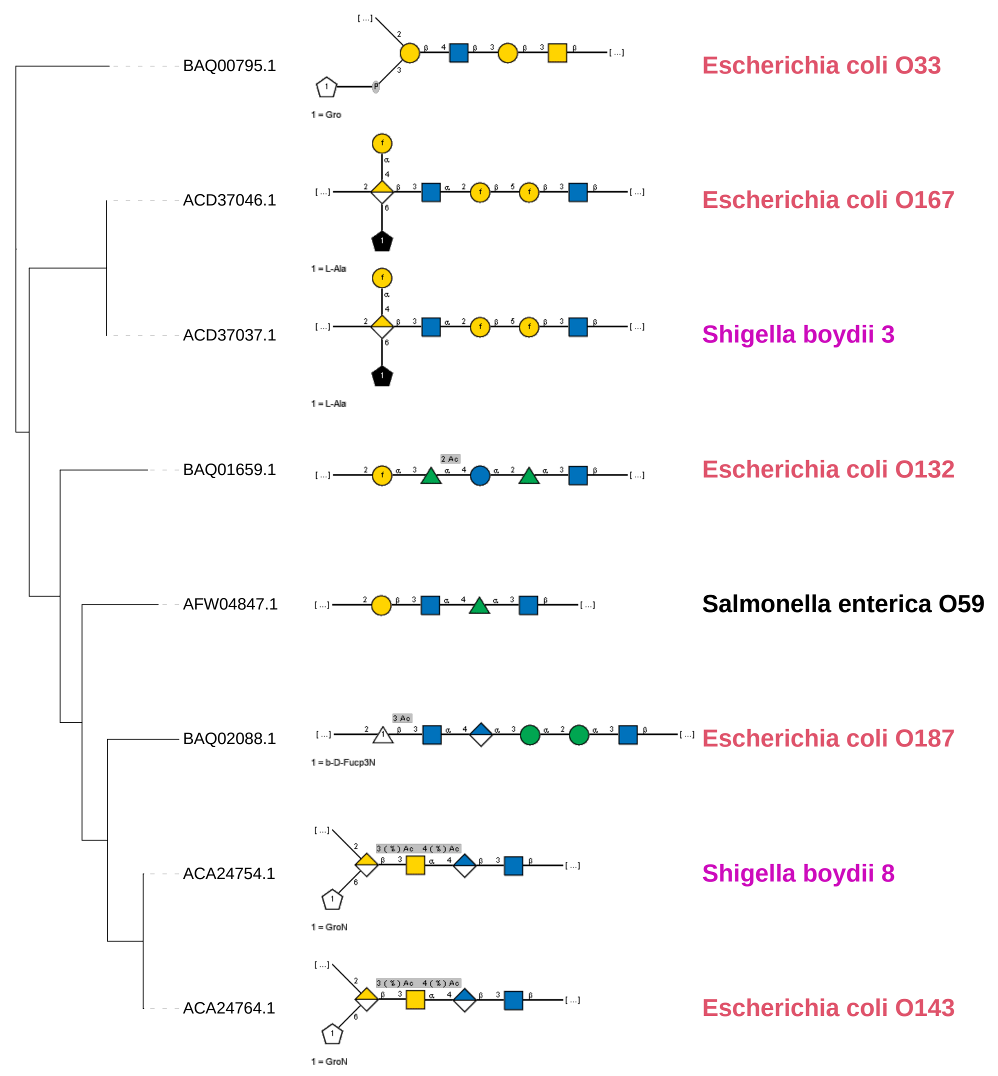

GT130:

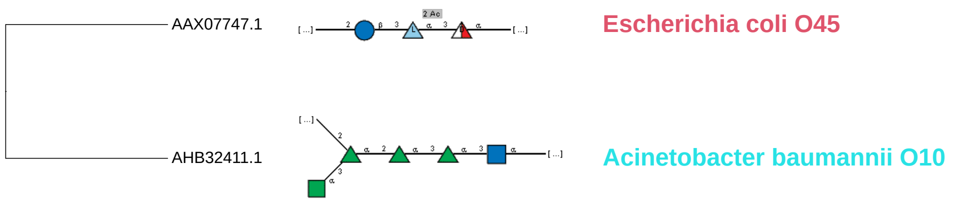

Supplementary Figure 4 – continued.

GT131:

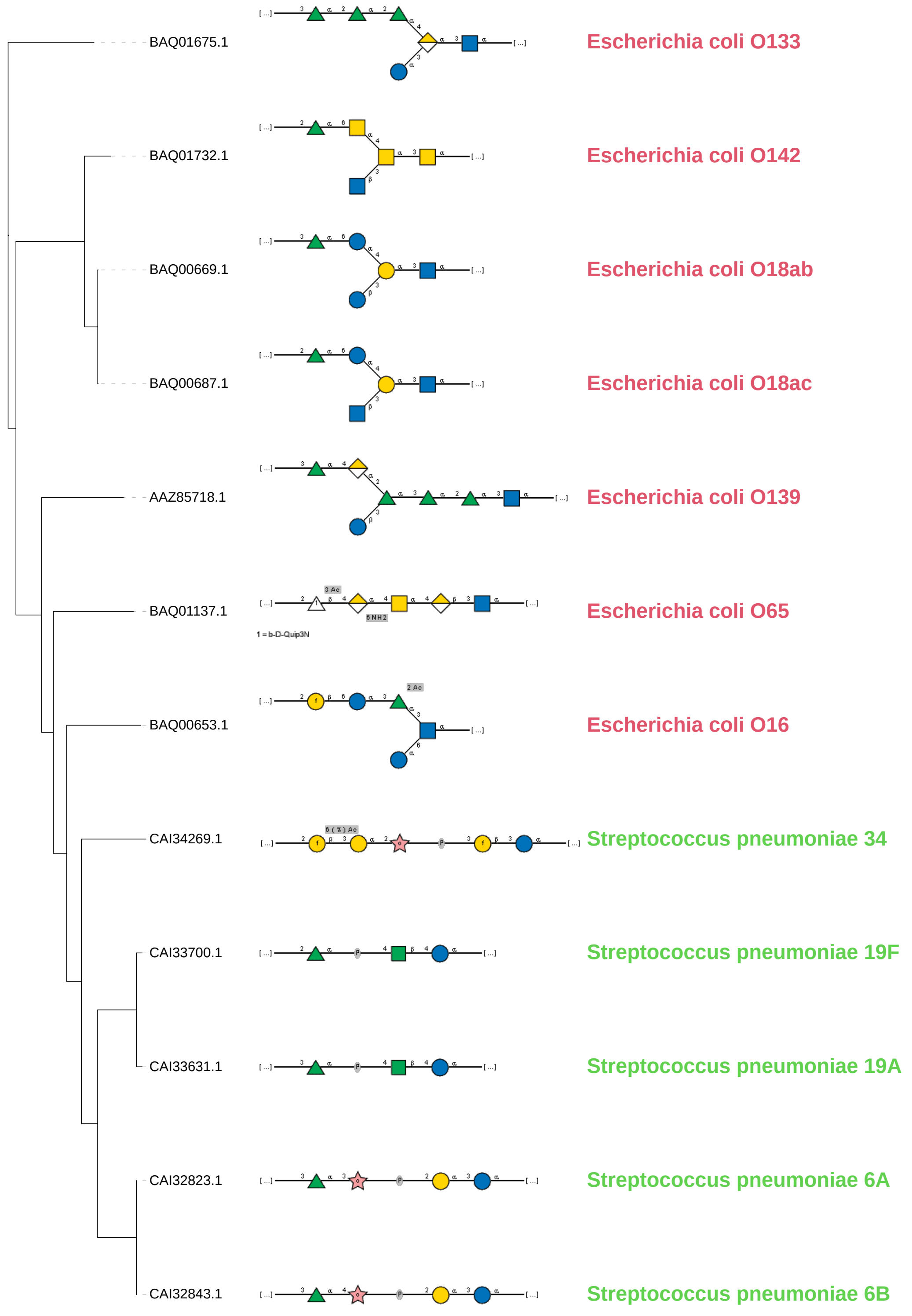

Supplementary Figure 4 – continued.

GT132:

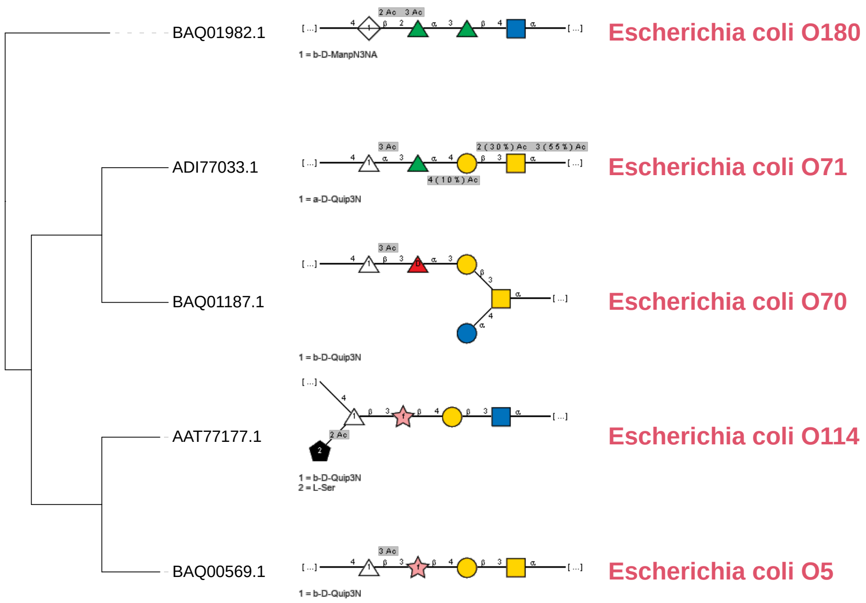

GT133:

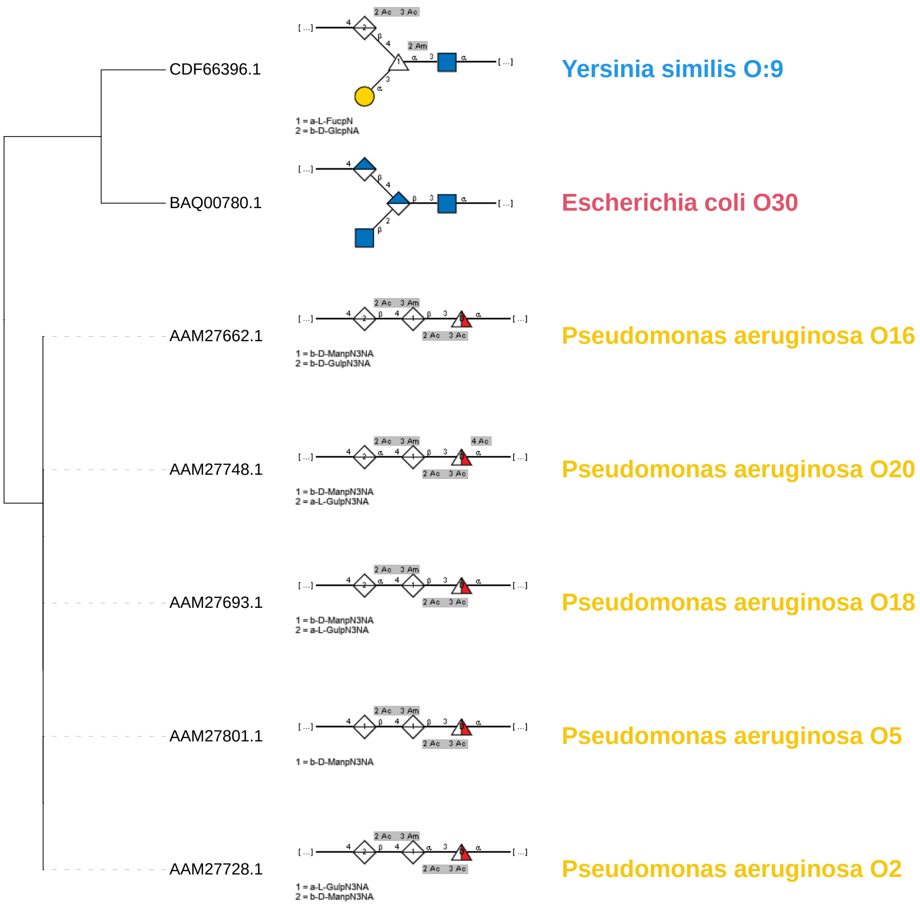

Supplementary Figure 4 – continued.

GT134:

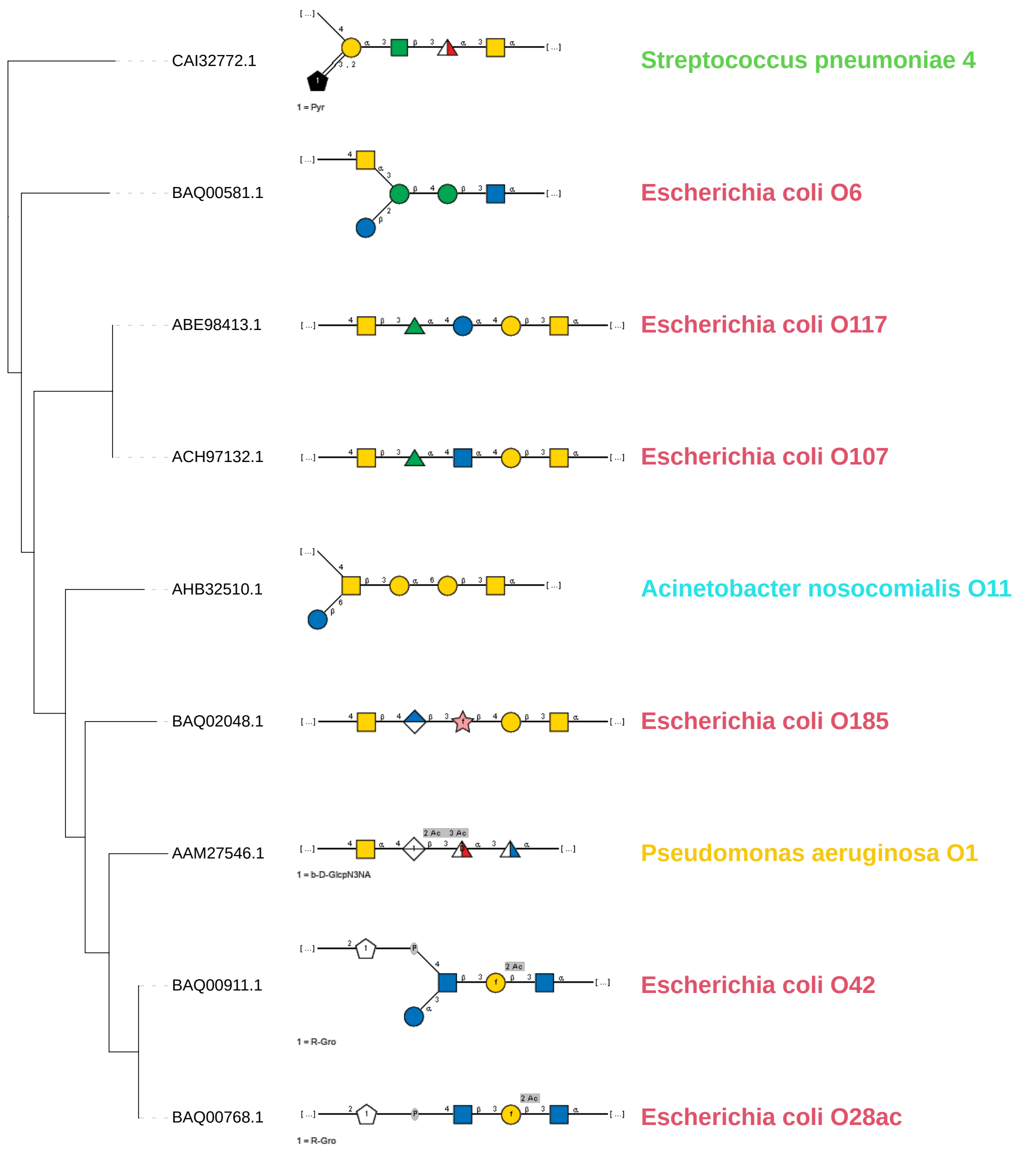

Supplementary Figure 4 – continued.

GT135:

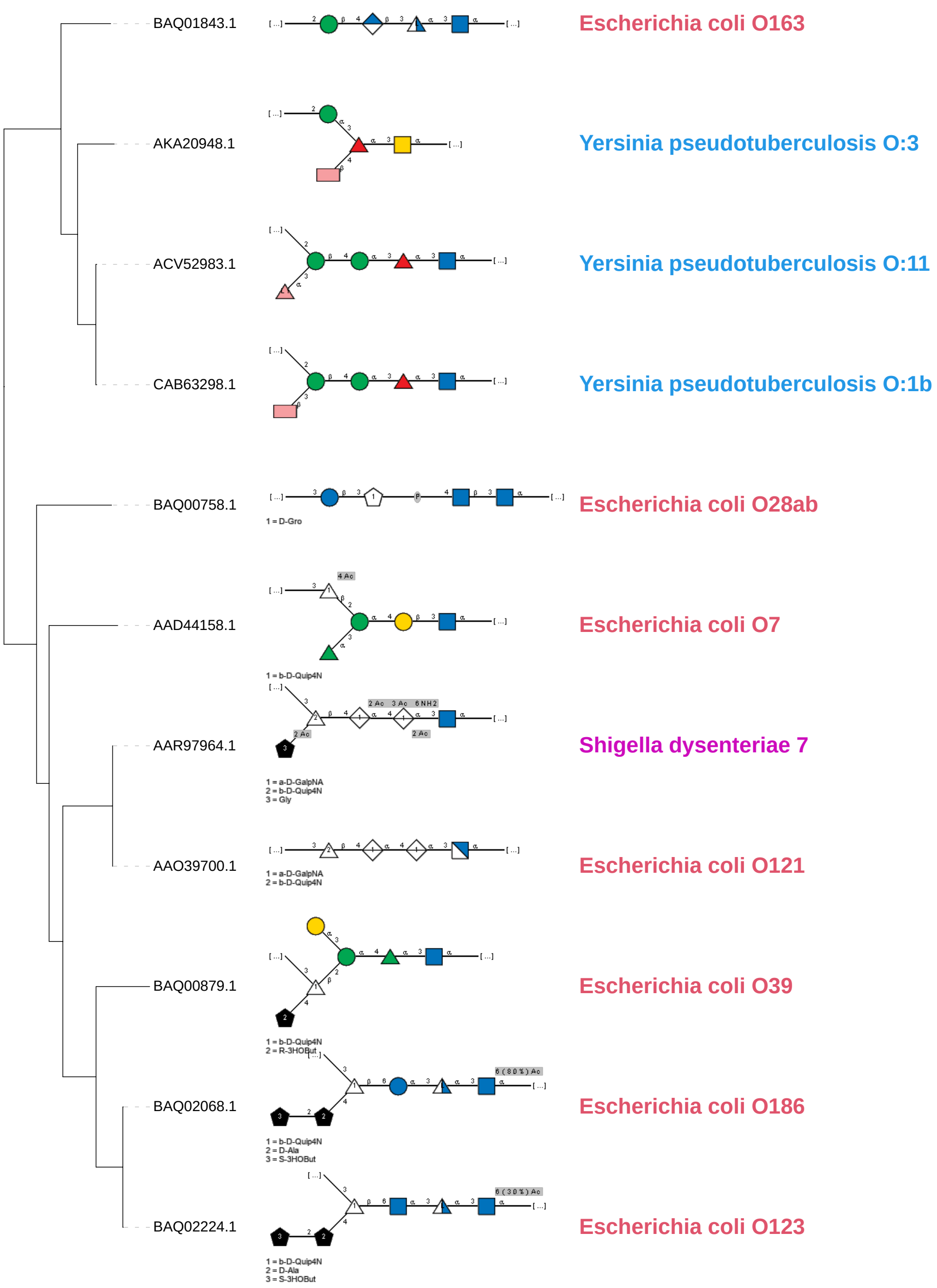

Supplementary Figure 4 – continued.

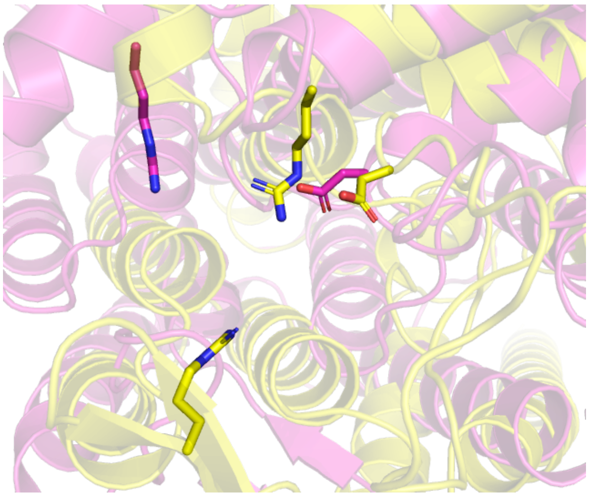

6BAR from GT119 (pink) and CAI34369 from GT125 (yellow). RMSD 5.7Å over 128 residues.

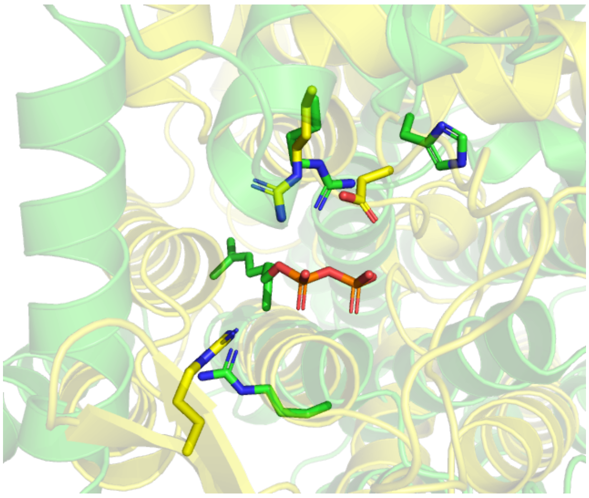

CAI34369 from GT125 (yellow) and 7TPG from GT121 (green). RMSD: 5.8Å over 160 residues.

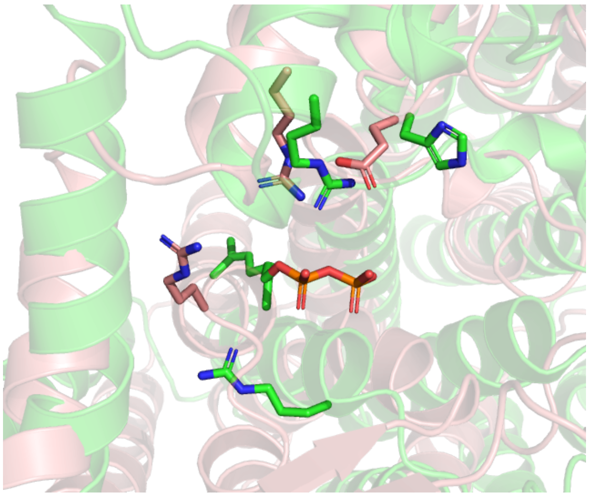

7TPG from GT121 (green) and AAM27615.1 from GT126 (pink). RMSD: 5.3Å over 192 residues

Supplementary Figure 5. Structural superimpositions of representative members from each family in clan GT-C_1. All structures are AlphaFold models except for 7TPG.

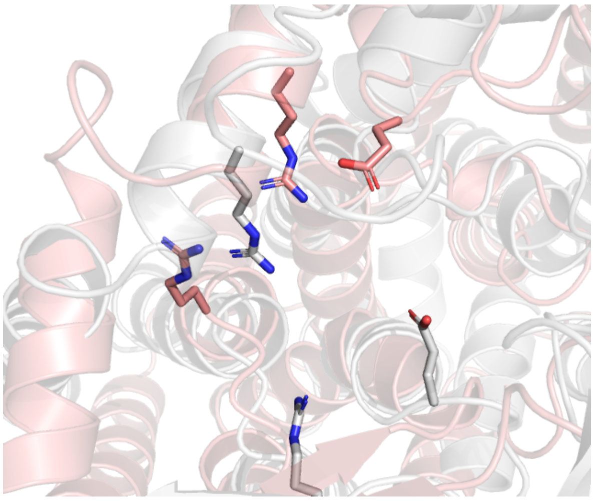

AAM27615.1 from GT126 (pink) and CAI34254.1 from GT127 (grey). RMSD: 5.4Å over 232 residues.

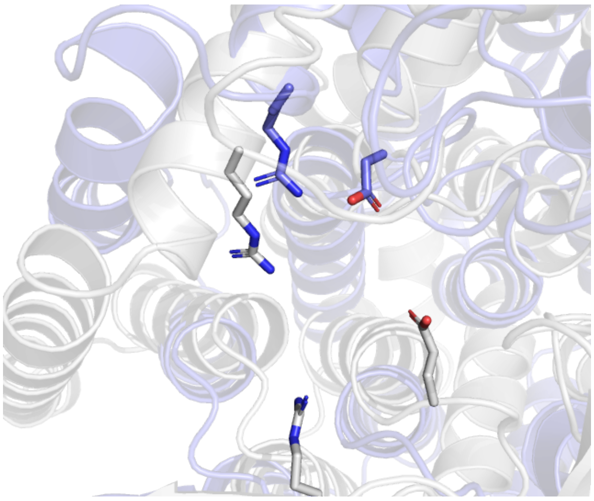

CAI34254.1 from GT127 (grey) and CAI34124.1 from GT128 (blue). RMSD: 5.7Å over 208 residues.

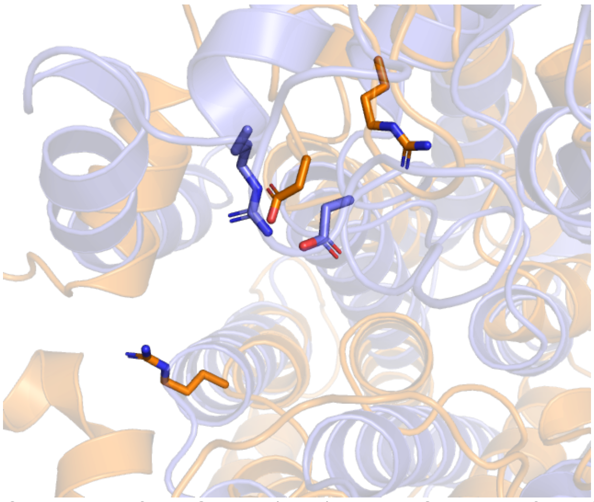

CAI34124.1 from GT128 (blue) and BAQ02088.1 from GT129 (orange). RMSD: 5.9Å over 144 residues.

Supplementary Figure 5 – continued.

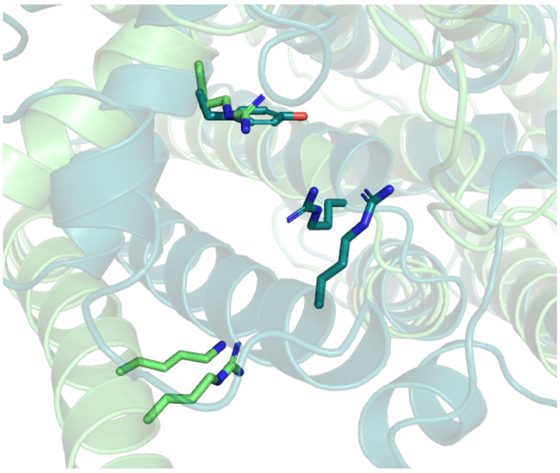

BAQ02224.1 from GT135 (green) and AHB32411.1 from GT130 (teal). RMSD: 4.9Å over 168 residues.

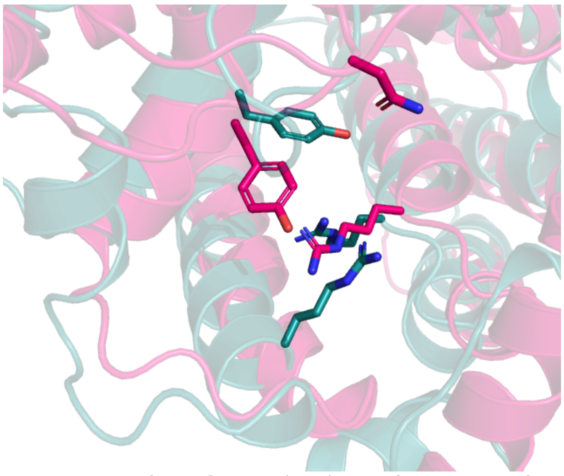

AHB32411.1 from GT130 (teal) and CAI32823.1 from GT131 (red). RMSD: 5.2Å over 328 residues.

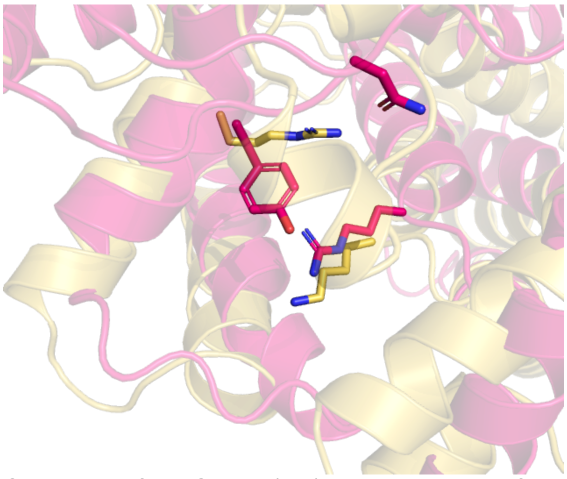

CAI32823.1 from GT131 (red) and AAT77177.1 from GT132 (yellow). RMSD: 4.9Å over 232 residues.

Supplementary Figure 6. Structural superimpositions of representative members from each family in clan GT-C_{2}. All structures are AlphaFold models.

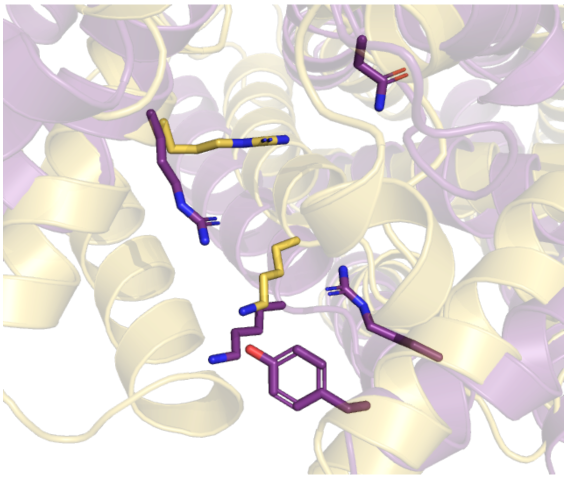

AAT77177.1 from GT132 (yellow) and AAM27801.1 from GT133 (purple). RMSD: 4.7Å over 200 residues.

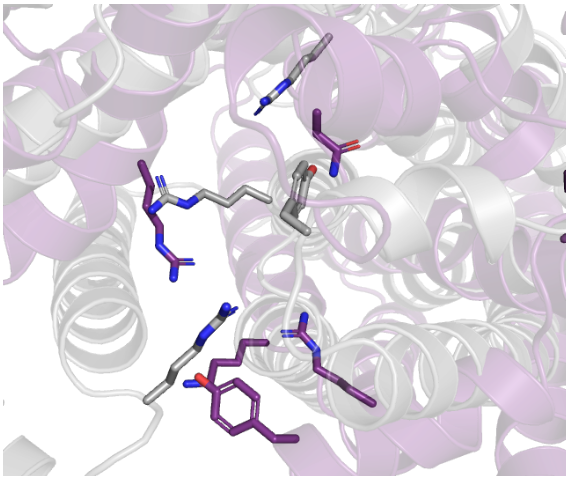

AAM27801.1 from GT133 (purple) and CAI32772.1 from GT134 (grey). RMSD: 5.8Å over 184 residues.

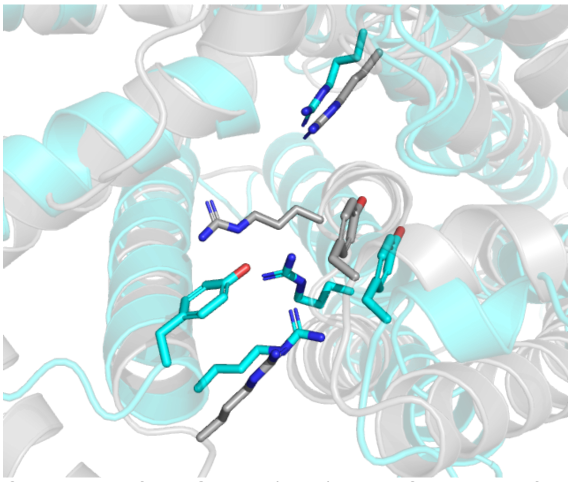

CAI32772.1 from GT134 (grey) and ACH50550.1 from GT120 (turquoise). RMSD: 5.5Å over 360 residues.

Supplementary Figure 6 – continued.

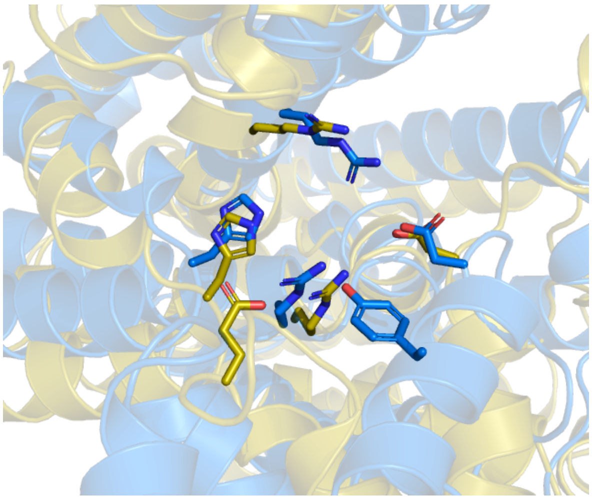

AHB32861.1 from GT122 (blue) and BAQ01641.1 from GT123 (gold). RMSD: 4.4Å over 200 residues.

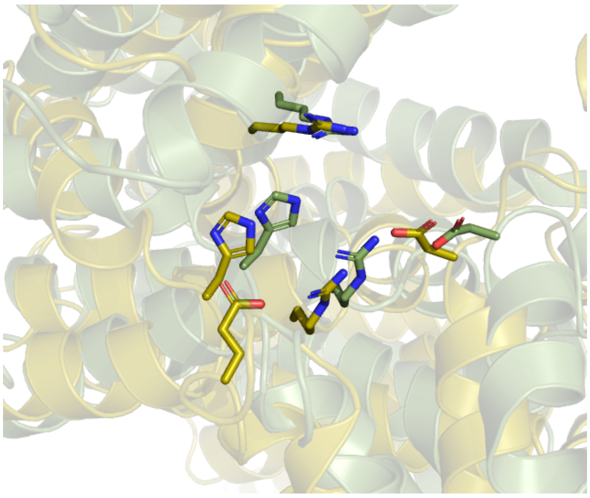

BAQ01641.1 from GT123 (gold) and ADI43271.1 from GT124 (green). RMSD 5.7Å over 352 residues.

Supplementary Figure 7. Structural superimpositions of representative members from each family in clan GT-C_3. All structures are AlphaFold models.
