# Supplementary material for: Diversity of sugar-diphospholipid-utilizing glycosyltransferase families
Source: Commun Biol. 2024 Mar 7;7:285. doi: 10.1038/s42003-024-05930-2 (PMC10920833; doi:10.1038/s42003-024-05930-2)
Supplement: Supplementary file 2 — Description of Supplementary Materials [file 42003_2024_5930_MOESM2_ESM.docx]

**Description of Additional Supplementary Files**

**File name:** Supplementary Data 1

**Description:** O-Lig seeds. List of seed sequences used for creation of the O-Lig family.

**File name:** Supplementary Table 2

**Description:** BP-Pol seed. List of BP-Pol sequences that were considered in this work
